# Differential Age-Dependent Import Regulation by Signal Peptides

**DOI:** 10.1371/journal.pbio.1001416

**Published:** 2012-10-30

**Authors:** Yi-Shan Teng, Po-Ting Chan, Hsou-min Li

**Affiliations:** 1 Institute of Molecular Biology, Academia Sinica, Taipei, Taiwan; 2 Graduate Institute of Life Sciences, National Defense Medical Center, Taipei, Taiwan; 3 Institute of Molecular and Cellular Biology, College of Life Science, National Taiwan University, Taipei, Taiwan; University of California San Francisco, United States of America

## Abstract

Gene-specific, age-dependent regulations are common at the transcriptional and translational levels, while protein transport into organelles is generally thought to be constitutive. Here we report a new level of differential age-dependent regulation and show that chloroplast proteins are divided into three age-selective groups: group I proteins have a higher import efficiency into younger chloroplasts, import of group II proteins is nearly independent of chloroplast age, and group III proteins are preferentially imported into older chloroplasts. The age-selective signal is located within the transit peptide of each protein. A group III protein with its transit peptide replaced by a group I transit peptide failed to complement its own mutation. Two consecutive positive charges define the necessary motif in group III signals for older chloroplast preference. We further show that different members of a gene family often belong to different age-selective groups because of sequence differences in their transit peptides. These results indicate that organelle-targeting signal peptides are part of cells' differential age-dependent regulation networks. The sequence diversity of some organelle-targeting peptides is not a result of the lack of selection pressure but has evolved to mediate regulation.

## Abstract 2

The import of certain proteins into chloroplasts is dependent on the age of the organelle, with age-dependent import being controlled by a specific motif within the signal peptide.

## Author Summary

It is well known that some genes are preferentially transcribed in young tissues and others are specifically expressed in aging tissue, but protein import into organelles is generally thought to be constitutive and independent of age. In this study, we find that, contrary to expectation, in higher plants the import of proteins into chloroplasts is indeed dependent on the age of the organelle. We find that chloroplast precursor proteins can be divided into three age-selective groups, with each having a preference for chloroplasts of a different age. The age-selective signal is located within the signal peptide of each protein that controls organelle import, and we further identify a motif that is necessary to make a signal peptide target older chloroplasts preferentially. We show that different members of a gene family often belong to different age-selective groups, and that changing the sequence motifs within a protein's signal peptide can change its age selectivity. These results indicate that organelle-targeting signal peptides are one set of tools available to multicellular organisms for differential age-specific regulation.

## Introduction

Gene-specific, age-dependent regulation is critical for the growth and development of all organisms. The regulation is known to take place at the transcriptional and translational levels. For example, some genes are preferentially transcribed, and some mRNAs or proteins are preferentially degraded, in younger or older tissues. In contrast, the process of protein import into organelles is generally thought to be constitutive. To our knowledge, it has not been previously shown that a specific group of proteins is preferentially imported into an organelle of a certain age. However, whether protein-specific, age-dependent regulation can exist at the level of organelle protein import has not been thoroughly investigated because of difficulties in isolating organelles of different ages.

Proteins imported into the endoplasmic reticulum (ER), mitochondria, and chloroplasts are usually synthesized as higher molecular weight precursors containing N-terminal signal peptides for organelle targeting. Another common feature of these three organelles is the high sequence heterogeneity of their signal peptides. Even among members of a gene family, the sequences of their signal peptides are often diverse, while their mature protein regions are highly similar. Consensus secondary-structure motifs have been identified for ER and mitochondrial signal peptides, but their signal peptides still vary greatly in sequences and lengths [1],[2]. No consensus sequence or structure has been identified for chloroplast-targeting signal peptides (herein called “transit peptides”), and their length varies from 13 to 146 amino acids [3],[4]. The reason for the sequence heterogeneity of these targeting signals is unknown. For the ER-targeting signal peptides, it has been proposed that the heterogeneity may have regulatory functions. Different sequences may interact with different accessory factors around the Sec61 translocon and thus modulate transport efficiency [5].

Protein import into chloroplasts is mediated by a translocon complex with components located in and around the two membranes of the envelope. Translocon components in the outer envelope membrane are called Toc (translocon at the outer-envelope-membrane of chloroplasts) proteins, and those in the inner envelope membrane are called Tic (translocon at the inner-envelope-membrane of chloroplasts) proteins (for reviews see [4],[6],[7]). By importing three representative proteins into chloroplasts of different ages, Dahlin and Cline showed that very young developing chloroplasts exhibited very high import efficiency; as chloroplasts matured, their import efficiency for all three proteins decreased dramatically [8]. These findings have led to the supposition that chloroplast protein import capability is regulated globally in concert with chloroplast protein demand. As chloroplasts mature, their protein demand decreases, and their import capacity for all proteins declines accordingly.

We show here that in higher plants, chloroplast precursor proteins can be divided into three age-selective groups, with each having a preference for chloroplasts of a different age. This differential age-dependent regulation is important for growth. We further define a necessary transit peptide motif for older chloroplast preference and show that different gene family members, through variations in their transit peptides, are preferentially imported into chloroplasts of different ages. This result suggests that one of the reasons for transit peptide sequence diversity among isoforms of a gene family is to achieve differential age-dependent import regulation.

## Results

### Precursor Proteins Can Be Classified into Three Age-Selective Groups

**Figure 1:**
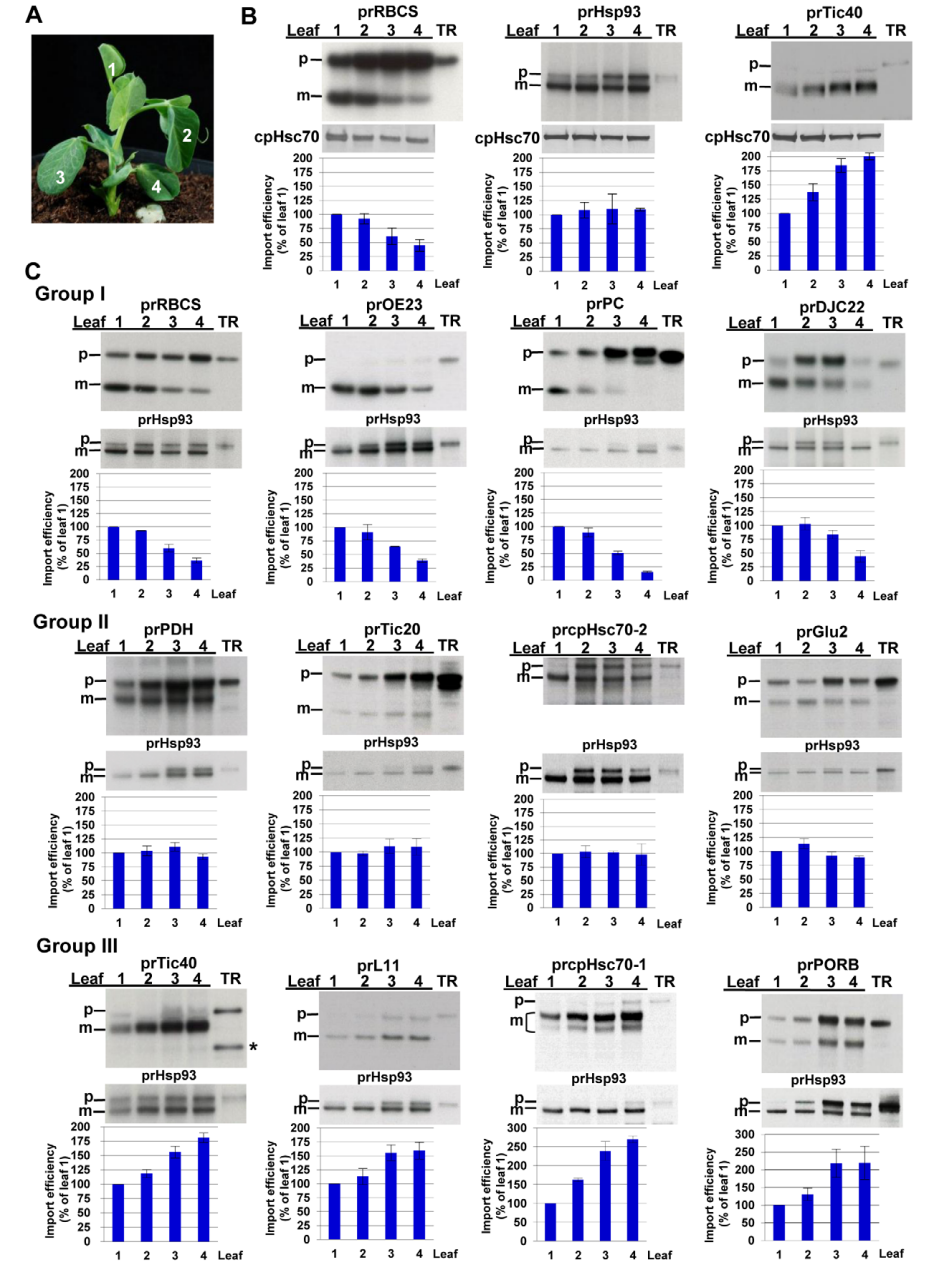
Chloroplast precursors are divided into three age-selective groups. (A) 16- to 18-d-old pea seedlings with four leaves and the youngest leaf still folded were used for chloroplast isolation. Leaf number designation is as labeled. (B) prRBCS, prHsp93, and prTic40 have different age preferences. Chloroplasts were isolated from leaves of different ages as shown in (A) and used for import experiments. Intact chloroplasts were re-isolated after the import reaction and analyzed by SDS-PAGE and autoradiography. An equal number of chloroplasts were loaded in each lane of the same gel. The amount of chloroplast Hsp70 (cpHsc70) from the same sample was analyzed by immunoblotting and used for normalization in the quantification shown in the bar graph below each gel. (C) Various precursor proteins were imported into chloroplasts isolated from pea leaves of different ages. Intact chloroplasts were re-isolated after the import reaction and analyzed by SDS-PAGE and autoradiography. The amount of mature Hsp93 imported in the same sample was used for normalization in quantifications shown in the bar graph below each gel. The amount of mature proteins imported in leaf 1 was set as 100%. Import of prcpHsc70-1 produces mature proteins of two different sizes [17],[50]. In vitro–translated prTic40 often produced two or three protein bands. The highest molecular weight protein is the true precursor. The lower molecular weight proteins (asterisk) are most likely produced by internal translation initiation or degradation and cannot bind to chloroplasts (data not shown). prTic40 in the import samples migrates right below the extremely abundant large subunit of RuBP carboxylase (RBCL) protein and thus appears to migrate faster than prTic40 in the TR lanes. Data shown are means ± standard deviation (SD), *n* = 3. m, mature form; p, precursor form; TR, in vitro–translated precursor proteins before import.

Chloroplasts isolated from different leaves, from top to bottom along the stem of pea seedlings, show increasing degrees of maturity [8]. Previously, when these chloroplasts were used to investigate the import of precursors to the small subunit of RuBP carboxylase (RBCS), light-harvesting complex of photosystem II (CAB), and small heat shock protein Hsp21, younger chloroplasts exhibited higher protein import efficiency [8]. We were interested in knowing the import behavior of some of the recently identified translocon components. Chloroplasts isolated from the four leaves of 16- to 18-d-old pea seedlings (the youngest leaf is designated as leaf number 1; Figure 1A) were tested for their ability to import prHsp93 (“pr” prefix for precursor forms; see Table S1 for full names and accession numbers of all precursors used) and prTic40, with prRBCS as a control. The level of endogenous chloroplast stromal Hsp70 (cpHsc70), which remains unchanged throughout the developmental stages tested [8] (Figure S1A), was analyzed by immunoblotting as a loading control. The import efficiency of prRBCS indeed declined as chloroplasts aged (Figure 1B). Surprisingly, prHsp93 showed similar import efficiency into chloroplasts isolated from all four leaves, and the import efficiency of prTic40 increased in older chloroplasts (Figure 1B).

We then tested more precursors (Table S1). For better quantification, prHsp93 was co-imported with each precursor, and import results were normalized to the amount of mature Hsp93 imported. The amount of precursor protein synthesized by the in vitro translation system is very small, so this co-import is unlikely to affect the import of other precursors by saturating the import machinery. Indeed, the age dependency of prRBCS and prTic40 did not change when results were normalized to Hsp93 (Figure 1C). However, if prHsp93 was not suitable for co-import for technical reasons (see Materials and Methods), endogenous cpHsc70 was still analyzed and used for normalization. Our results showed that precursors could be divided into at least three age-selective groups. Group I precursors, which include prRBCS, prOE23, prPC, prDJC22 (Figure 1C), prDJC75, prOE33, and prFd-protA (Figure S2), were preferentially imported into younger chloroplasts. Group II precursors, which include prHsp93, prPDH, prTic20, prcpHsc70-2, and prGlu2 (Figure 1C), were imported into chloroplasts of different ages with similar efficiency. Group III precursors, which include prTic40, prL11, prcpHsc70-1, and prPORB (Figure 1C), exhibited increasing import efficiency as chloroplasts aged. The separation of precursors into three age-selective groups was observed when import was performed with an equal number of plastids or an equal amount of chlorophylls in each reaction (see Materials and Methods and Figure S3). Although most of the precursors we used were from *Arabidopsis*, some of them were from other species (Table S1), including three from pea (prPC, prHsp93, and prTic40). We have also tested the import of rice and *Arabidopsis* prTic40 and *Physcomitrella patens* prOE23 and prL11. Their age selectivity was the same as that of their orthologs (data not shown). Thus, it is unlikely that the different import patterns we observed were due to the species origin of the precursors.

### The Age-Selective Signal Is Located in the Transit Peptide

**Figure 2:**
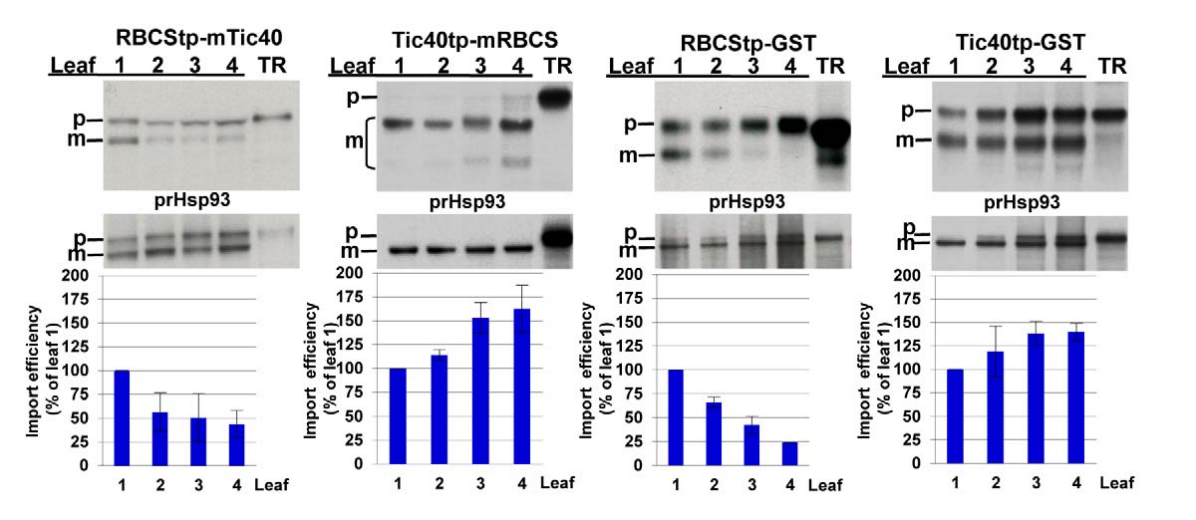
Age selectivity is determined by the transit peptide. Chimeric precursors consisting of swapped prRBCS and prTic40 transit peptides (RBCStp-mTic40 and Tic40tp-mRBCS), or prRBCS or prTic40 transit peptide fused to GST (RBCStp-GST and Tic40tp-GST), were co-imported with prHsp93 into chloroplasts of different ages. Chloroplasts from Tic40tp-mRBCS import experiments were further treated with thermolysin after the import reactions. The amount of mature Hsp93 imported in the same sample was used for normalization in quantifications shown in the bar graph below each gel. The amount of mature proteins imported in leaf 1 was set as 100%. Data shown are means ± SD, *n* = 3. m, mature form; p, precursor form; TR, in vitro–translated precursor proteins before import.

To investigate which part of a precursor protein was responsible for the age selectivity, we swapped the transit peptides between prRBCS (representative of group I precursors) and prTic40 (representative of group III precursors) and imported the fusion proteins, RBCStp-mTic40 (“tp” for transit peptide and “m” for mature protein) and Tic40tp-mRBCS, into chloroplasts isolated from leaves of different ages. RBCStp-mTic40 had an import pattern similar to that of prRBCS (Figure 2). Tic40tp-mRBCS produced two lower molecular weight proteins after import, most likely because the prTic40 transit peptide is processed twice [9],[10]. To make sure both proteins were within chloroplasts, after the import of Tic40tp-mRBCS, chloroplasts were treated with thermolysin to remove chloroplast-surface-bound proteins, and thermolysin-resistant mature proteins were quantified. The result showed that Tic40tp-mRBCS had age selectivity similar to that of prTic40 (Figure 2). We further fused the transit peptides of prRBCS and prTic40 to the N terminus of GST and imported the fusion proteins into chloroplasts of different ages. RBCStp-GST was preferentially imported into younger chloroplasts, just like prRBCS, and Tic40tp-GST was preferentially imported into older chloroplasts, just like prTic40 (Figure 2). Therefore, transit peptides are sufficient to determine age selectivity.

**Figure 3:**
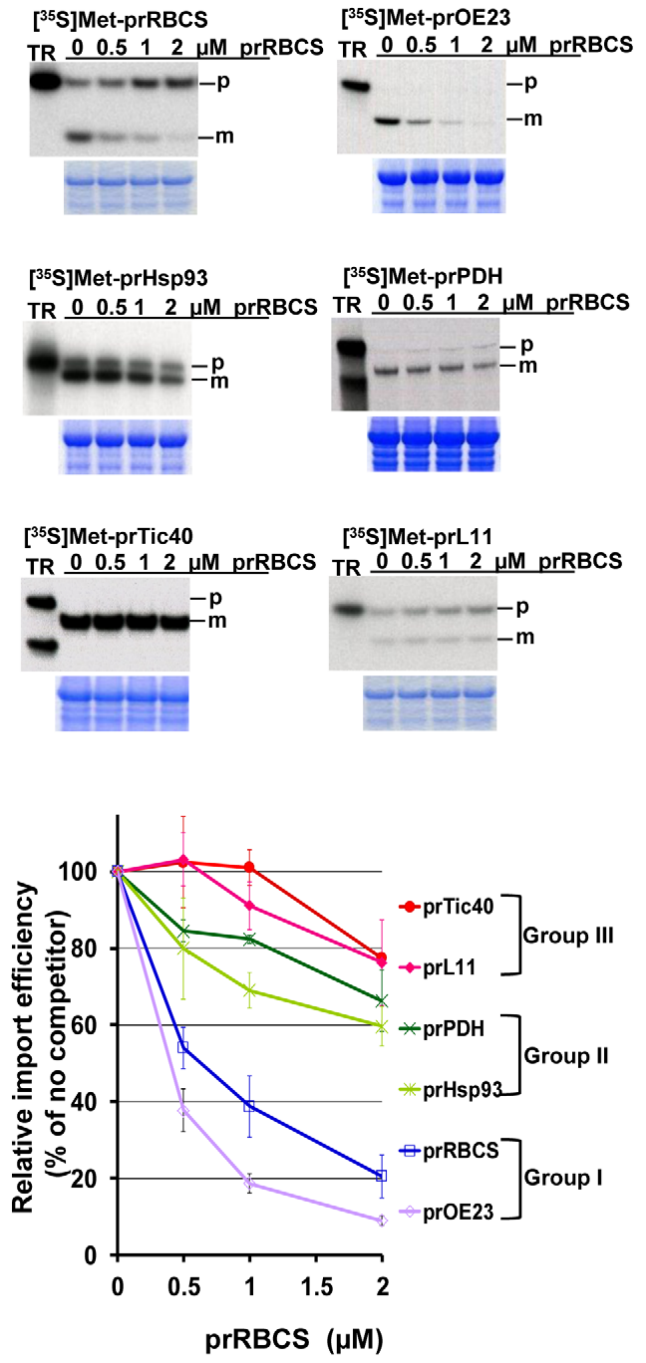
Import competition of a group I precursor with representative precursors of the three groups. Various [^35^S]Met-labeled precursors were imported with 0 to 2 µM recombinant prRBCS, as labeled on top of the gel, into chloroplasts isolated from 8-d-old pea seedlings for 15 min. Intact chloroplasts were re-isolated and analyzed by SDS-PAGE, stained with Coomassie Blue, and dried for autoradiography. An equal amount of protein was loaded in each lane of the same gel. The region around the endogenous RBCL is shown below the autoradiograph to confirm that loading was equal in each lane. Imported mature protein was quantified using a phosphorimager and normalized to the amount of endogenous RBCL obtained by scanning the Coomassie Blue–stained gels. The line graph shows quantifications of relative import efficiencies, with the amount of mature protein imported in the absence of recombinant prRBCS set as 100%. Precursor groups are labeled on the right. Data shown are means ± SD, *n* = 3. m, mature form; p, precursor form; TR, in vitro–translated precursor proteins before import.

If precursors are divided into three groups because of different characteristics of the transit peptides among different groups, then in import competition experiments, one precursor in excess should out-compete the import of precursors from the same group, but would have a smaller effect on the import of precursors from other groups. To test this we performed import competition experiments using *Escherichia coli*–produced recombinant prRBCS (a group I precursor), in vitro–translated [^35^S]Met-labeled representative precursors from all three groups and chloroplasts isolated from 8-d-old pea seedlings. The degree of competition was indeed divided into three groups (Figure 3). At 0.5 µM recombinant prRBCS, the import of group I precursors prRBCS and prOE23 decreased by 50% to 60%, import of group II precursors prHsp93 and prPDH deceased by 20%, while the import of group III precursors prTic40 and prL11 was not affected. At 2 µM recombinant prRBCS, the import of the two group I precursors decreased 80%, import of the two group II precursors decreased about 40%, and the import of the two group III precursors decreased about 20%. This result indicates that different precursors in the same age-selective group most likely share the same import pathway.

### Differential Age-Selective Import Is Physiologically Important

If the differential age-dependent regulation we observed is physiologically important, it could be expected that a group III precursor engineered with a group I transit peptide would not function optimally, which should have an impact on plant growth if the affected protein is important for growth. To test this idea, we examined the complementation of an *Arabidopsis tic40* knockout mutant by the hybrid protein RBCStp-mTic40, in which the group III prTic40 transit peptide was replaced by the group I prRBCS transit peptide. It has been shown that information for targeting and insertion of prTic40 to the inner membrane is contained within the mature region of prTic40 [9],[10], and replacing the prTic40 transit peptide with the prRBCS transit peptide did not affect the proper insertion and topology of Tic40 in the inner membrane [9]. We also first verified that the in vitro import efficiency of RBCStp-mTic40 and prTic40 into *Arabidopsis* chloroplasts was comparable (data not shown).

**Figure 4:**
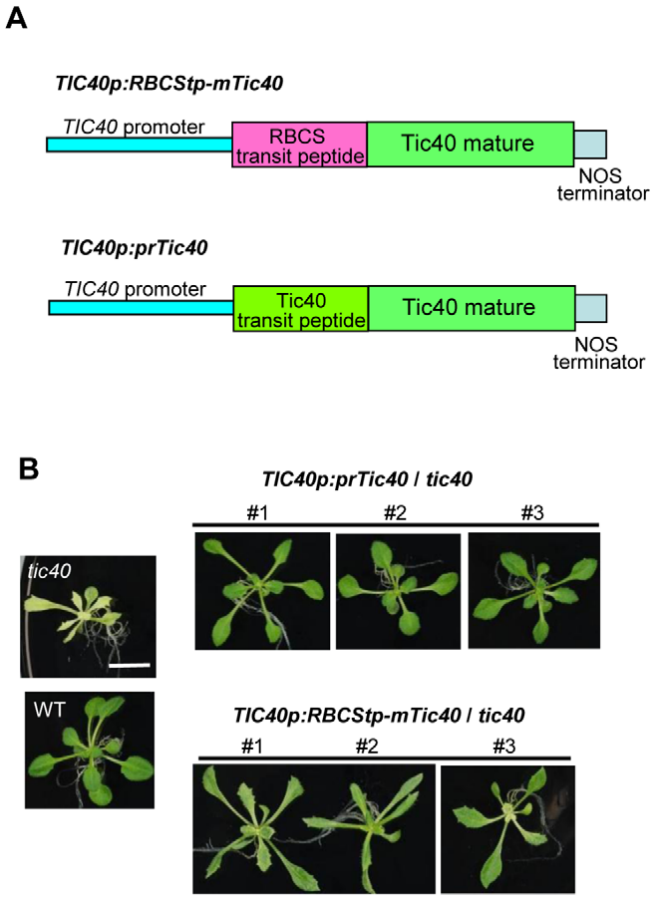
RBCStp-mTic40 cannot fully complement the *tic40* mutant. (A) Constructs used to transform the *Arabidopsis tic40* mutant. *RBCStp-mTic40* or *prTic40* cDNA was placed downstream of a 2-kb *TIC40* native promoter. (B) Phenotypes of transgenic plants. Three independent lines are shown for each transgene. All plants were grown on MS plates for 21 d. A wild-type (WT) plant and the original *tic40* mutant are shown on the left. All pictures are shown with the same magnification. Bar = 1 cm.

Tic40 is required for protein import into chloroplasts, and its knockout mutants have extremely retarded growth and small and pale green leaves with jagged leaf margins [11],[12]. The RBCStp-mTic40-encoding cDNA was placed under the control of a 2-kb promoter fragment from the *TIC40* gene (Figure 4A). The original prTic40-encoding cDNA under the control of the same *TIC40* promoter fragment was used as a control. The two constructs, designated as *TIC40p:RBCStp-mTic40* and *TIC40p:prTic40*, were used to transform *tic40-1*, a knockout mutant caused by T-DNA insertion in the *TIC40* gene [11]. As shown in Figure 4B, the *TIC40p:prTic40* transgenic plants were indistinguishable from the wild type, indicating that the *tic40* mutation was fully complemented. In contrast, the *TIC40p:RBCStp-mTic40* transgenic plants all had a variable pale green color in between that of the *tic40* mutant and the wild type, and retained the jagged-leaf-margin phenotype of the *tic40* mutant, even though the steady state Tic40 protein level in the mature plants was comparable to that in the wild-type and in the *TIC40p:prTic40*-complemented plants (Figure S4A). Fractionation of isolated chloroplasts also showed that Tic40 proteins were localized in the inner envelope membrane in both transgenic plants (Figure S4B). These results indicate that replacing the prTic40 transit peptide with the prRBCS transit peptide prevented Tic40 from functioning optimally. However, we cannot exclude the possibility that there are differences other than age selectivity between the prRBCS and prTic40 transit peptides. For example, studies have shown that prRBCS cannot be imported into isolate pea root plastids [13], although in stable transgenic wheat and maize lines, the prRBCS transit peptide can direct fusion protein import into plastids in root hair cells [14],[15]. Thus, it is possible that the incomplete complementation is caused by failure of prRBCS transit peptide to direct Tic40 import into other cell types in roots.

### Protein Import into *Chlamydomonas* Chloroplasts Does Not Show Differential Regulation

To begin to understand the physiological reason for the observed differential age-dependent import, we investigated whether a similar regulation exists in unicellular organisms. We used the unicellular green alga *Chlamydomonas reinhardtii*. Each *Chlamydomonas* cell contains a single chloroplast. Furthermore, cultures of *Chlamydomonas* can be synchronized by light/dark cycles, and ages of chloroplasts can be defined by hours of culturing in the light [16]. Thus, *Chlamydomonas* also has an age spectrum of chloroplasts during development but does not face the problem of having leaves/chloroplasts of different ages supported on the same stem in the same organism. If group III precursors prefer older chloroplasts solely because of chloroplast maturity, then group III precursors should also prefer to be imported into *Chlamydomonas* chloroplasts isolated from cells that have been cultured for longer periods in the light.

Chloroplasts isolated from synchronized *Chlamydomonas* cells cultured for 1, 6, and 11 h in the light were used for in vitro import experiments. Endogenous Hsp70B of *Chlamydomonas* chloroplasts analyzed by immunoblots was used as a loading control, as Hsp70B level was steady in the three time points analyzed (Figure S1B), similar to cpHsc70 in pea chloroplasts. We tested the import of one group I precursor (prOE23) and two group III precursors (prTic40 and prL11). We also obtained cDNA encoding *Chlamydomonas* chloroplast precursor proteins Cr-prRBCS and Cr-prL11 (prefix “Cr” for *C. reinhardtii*) for import experiments.

**Figure 5:**
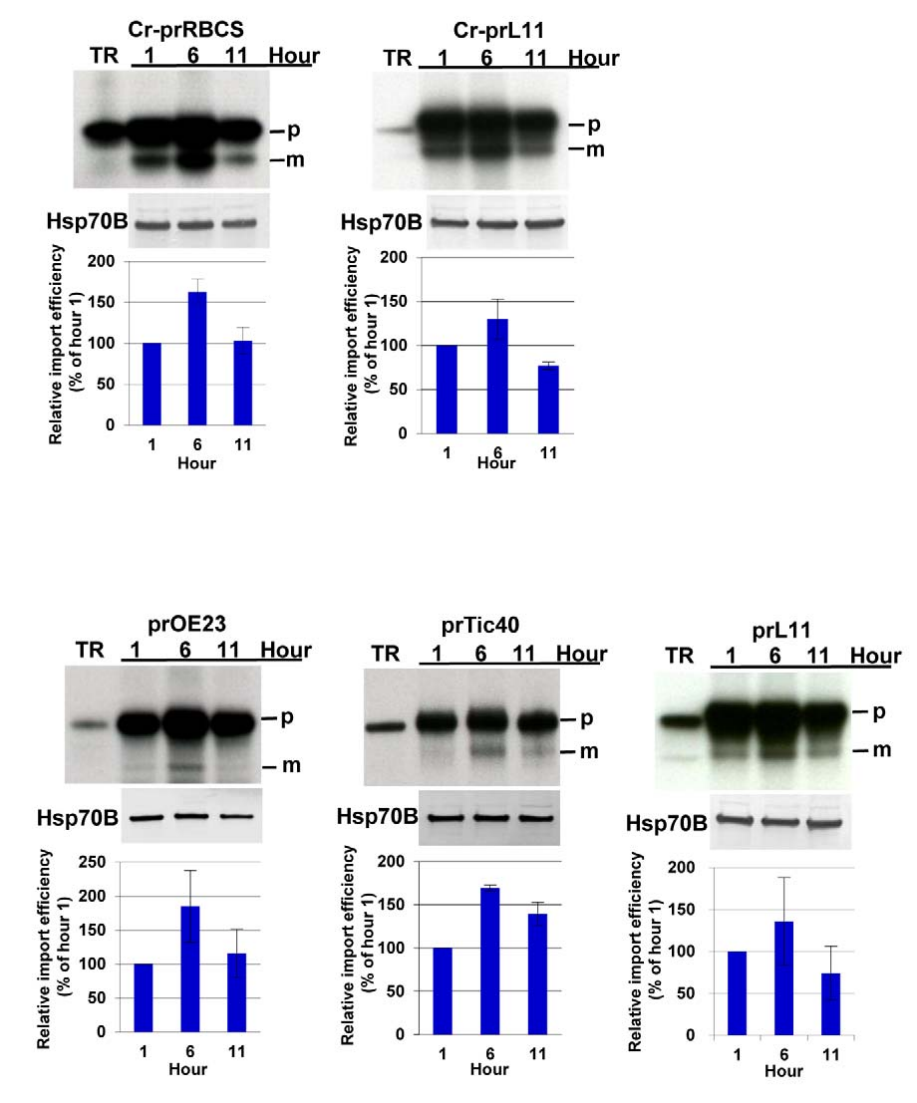
Protein import into *Chlamydomonas* chloroplasts is not differentially regulated. [^35^S]Met-labeled precursors of Cr-prRBCS, Cr-prL11, prOE23, prTic40, and prL11 were imported into chloroplasts isolated from synchronized cultures of *Chlamydomonas* 1, 6, and 11 h after the start of the third light cycle and used for import experiments. Samples were analyzed by SDS-PAGE and autoradiography. An equal number of chloroplasts were loaded in each lane of the same gel. The amount of *Chlamydomonas* chloroplast Hsp70B was analyzed by immunoblotting and used for normalization of quantifications in the bar graph shown below each gel. The amount of mature protein imported at hour 1 was set as 100%. Data shown are mean ± SD, *n* = 3. m, mature form; p, precursor form; TR, in vitro–translated precursor proteins before import.

Similar to results reported previously [16], import of Cr-prRBCS was highest in the 6-h chloroplasts and lower in the 1- and 11-h chloroplasts (Figure 5). All four other precursors tested showed the same pattern as Cr-prRBCS, despite the fact that they were recognized as group I (prOE23 and Cr-prRBCS) and group III precursors (prTic40, prL11, and Cr-prL11) by pea chloroplasts (Figures 1 and S5). These results suggest that protein import into *Chlamydomonas* chloroplasts shows only a global regulation for all proteins, rather than a differential regulation according to precursor groups like in higher plants.

### Two Consecutive Positive Charges Is a Necessary Motif for Group III Transit Peptides

**Figure 6:**
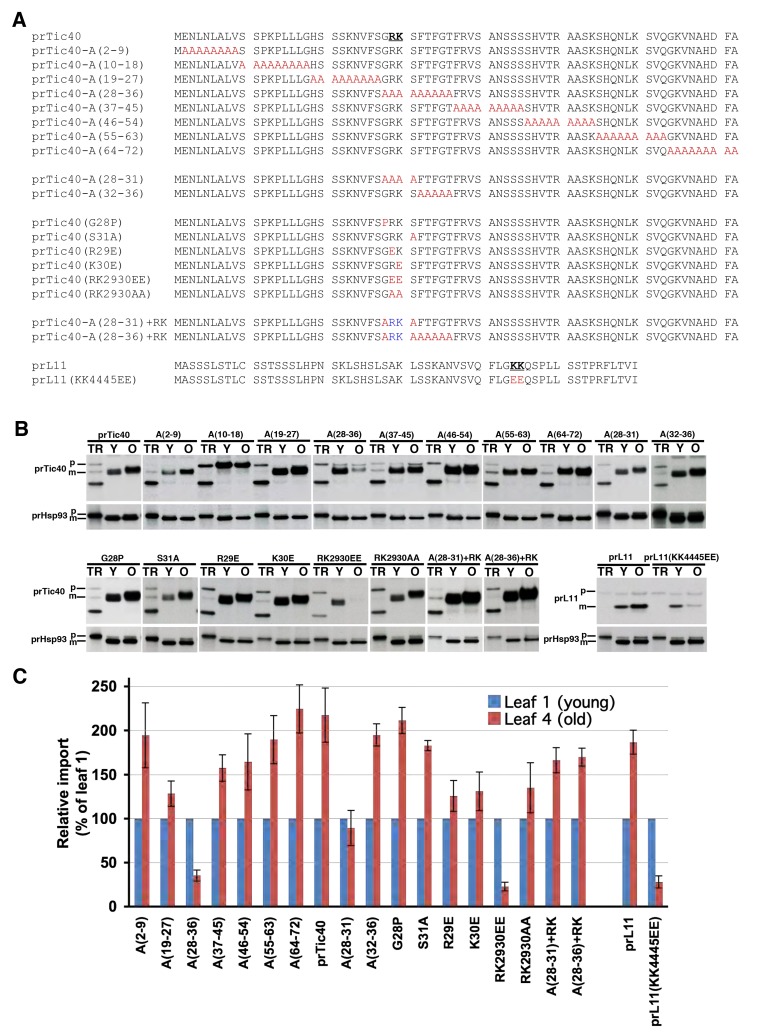
Two consecutive positive charges is a necessary motif for group III transit peptide preference of older chloroplasts. (A) Transit peptide sequences of prTic40, prL11, and their mutants. The two consecutive amino acids with positive charges are in bold and underlined, and the substitutions are in red and blue. (B) Import of prTic40, prL11, and their transit peptide mutants into young (Y, from leaf 1) and old (O, from leaf 4) chloroplasts. The prHsp93 import shown below each mutant was from the same sample (co-imported) run on the same gel. TR, in vitro–translated precursor proteins before import. (C) Quantification of import experiments shown in (B). For each precursor, the amount of mature protein imported was first normalized to the amount of Hsp93 imported in the same sample. The normalized amount in the leaf 1 chloroplasts was then set as 100%. No quantification data for the A(10–18) mutant of prTic40 is presented because no mature proteins were produced.

If precursors are divided into three groups because of different characteristics of their transit peptides, there should be some sequence motif that is the determining feature for each group of transit peptides. We started with the group III transit peptides because signals that prefer older organelles represent a novel finding of our current study and may have biotechnology applications. Not surprisingly, comparing the transit peptides of the group III precursors we had identified did not reveal any consensus sequence motif. We then used the transit peptide of prTic40 as a model and made a series of alanine substitution mutants to systematically identify the regions important for older chloroplast recognition. The 72-residue prTic40 transit peptide was divided into eight blocks of nine amino acids, and each block was replaced with nine Ala residues (Figure 6A). These Ala substitution mutants were imported into young and old chloroplasts isolated from the first and fourth leaves, respectively.

All mutants, with the exception of A(10–18) and A(28–36), still showed a preference for older chloroplasts (Figure 6B and 6C). No imported mature protein was observed for the A(10–18) mutant in either young or old chloroplasts. To determine the location of the A(10–18) mutant precursors, after import of A(10–18), chloroplasts were treated with trypsin, which can only penetrate the outer membrane but not the inner membrane. A(10–18) mutant precursors were completely degraded by trypsin, while imported wild-type mature Tic40 was resistant to trypsin digestion, indicating that the A(10–18) mutant precursors were outside the inner membrane (Figure S6A). These results suggest that the A(10–18) mutant could bind to chloroplasts but could not be translocated into the stroma. The A(10–18) mutations most likely affected interaction of the prTic40 transit peptide with some translocon components that are required for translocation into both young and old chloroplasts. Furthermore, in all mutants except A(10–18), we did not observe a significant increase in accumulation of precursors, suggesting that the mutations did not affect the processing of prTic40 transit peptide.

The A(28–36) mutation severely reduced the import into older chloroplasts, indicating that residues 28 to 36 are important for older chloroplast recognition (Figure 6B and 6C). These residues were further divided into two halves, and two more alanine substitution mutants were generated: A(28–31) and A(32–36). A(28–31) no longer preferred older chloroplasts, while the A(32–36) mutant still did (Figure 6B and 6C). Residues 28 to 31 (Gly28-Arg29-Lys30-Ser31) were further individually substituted with amino acids with characteristics different from those of the original amino acids. Substitution of Gly28 with Pro (Figure 6B and 6C) or His or Ala (data not shown), or substitution of Ser31 with Ala (Figure 6B and 6C), did not change the age selectivity of prTic40. In comparison, substitution of the positively charged Arg29 or Lys30 with Glu, or substitution of both residues with Ala, reduced preference for older chloroplasts. Substitution of both residues with Glu almost knocked out the import into older chloroplasts. Adding back Arg29 and Lys30 into the A(28–36) and A(28–31) mutants, which had severely reduced import into older chloroplasts, restored the preference of prTic40 for older chloroplasts (Figure 6B and 6C). These data indicate that the two consecutive positive charges of Arg29 and Lys30 are important components for older chloroplast recognition in the prTic40 transit peptide. We further confirmed this result using another group III precursor, prL11. Its transit peptide has one pair of consecutive positive charges, at residues 44 and 45. Mutating Lys44 and Lys45 to Glu (the prL11[KK4445EE] mutant) severely knocked down import into older chloroplasts without affecting import into younger chloroplasts (Figures 6B, 6C, and S6B), confirming that having two consecutive positive charges is a determining feature of group III transit peptides. However, two consecutive positive charges are also present in some group I and group II transit peptides. Thus, this motif is only one necessary feature. Other yet unknown structural motifs are required to work together with the two positive charges to build a sufficient group III signal.

### Isoforms of a Gene Family Are Preferentially Imported into Chloroplasts of Different Ages because of Differences in Transit Peptide Sequences

**Figure 7:**
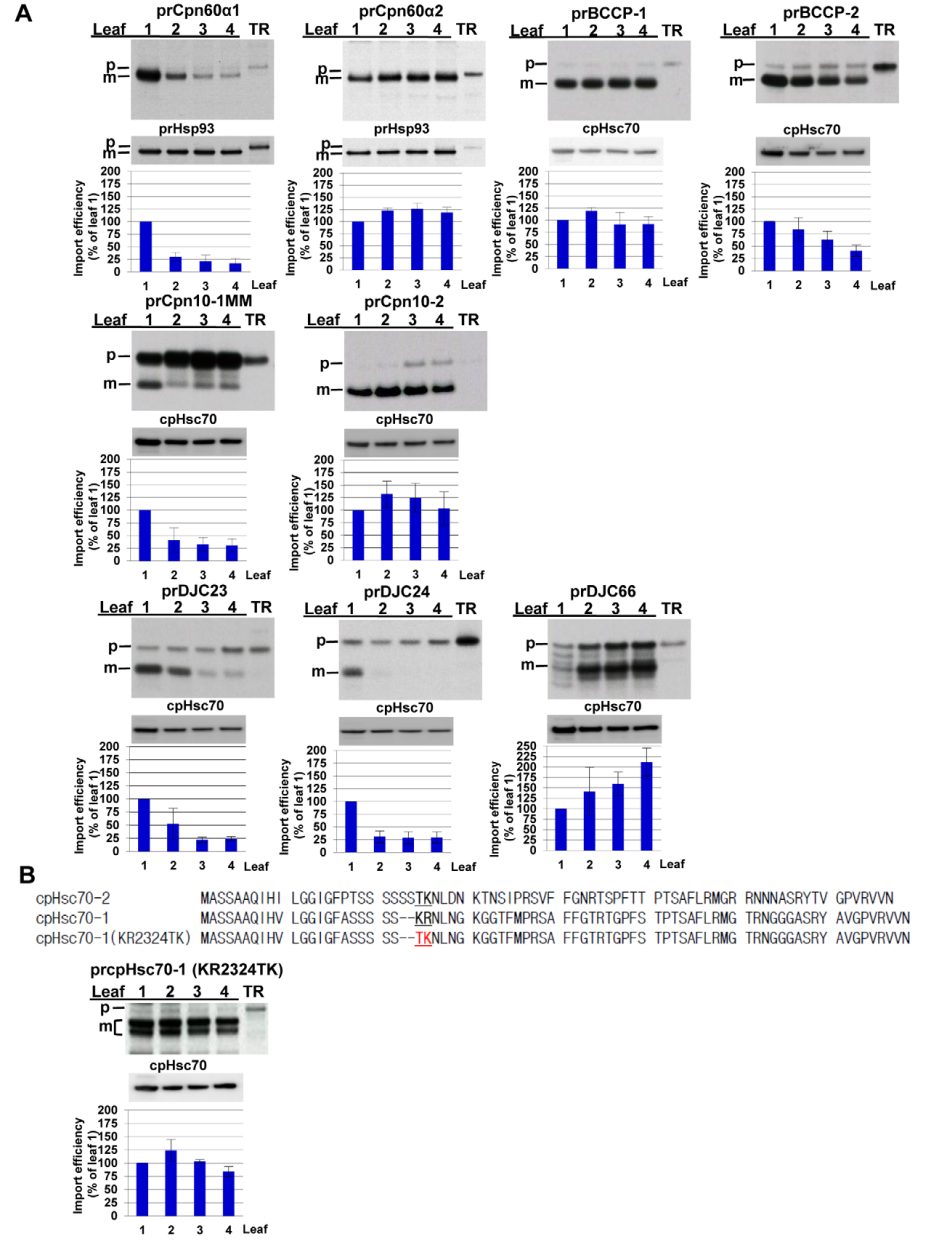
Different isoforms are preferentially imported into chloroplasts of different ages because of sequence differences in transit peptides. (A) Import of different isoforms of gene families. (B) Transit peptide sequences of prcpHsc70-2, prcpHsc70-1, and prcpHsc70-1(KR2324TK), and import of prcpHsc70-1(KR2324TK). Various precursor proteins were imported into chloroplasts isolated from pea leaves of different ages. Intact chloroplasts were re-isolated after the import reaction and analyzed by SDS-PAGE and autoradiography. The amount of endogenous cpHsc70 or co-imported Hsp93 of the same sample was analyzed and used for normalization in quantifications shown in the bar graph below each gel. The amount of mature proteins imported in leaf 1 was set as 100%. Data shown are means ± SD, *n* = 3. m, mature form; p, precursor form; TR, in vitro–translated precursor proteins before import.

Many genes have duplicated from single genes in unicellular organisms into gene families in multicellular organisms. This duplication is often attributed to the need for tissue-specific regulation at the transcriptional level. However, for gene families encoding organelle proteins, the organelle-targeting signals are often diverse, despite having highly similar mature regions. The reason for this high sequence heterogeneity in the targeting signals is not known. Among the chloroplast precursors we tested, cpHsc70-1 and cpHsc70-2 provide one such example (Figure S7A). While the mature regions of the two isoforms are 93% identical, the transit peptide region has only 75.5% identity. Interestingly, in green algae, including *Chlamydomonas*, chloroplast Hsp70 is encoded by a single gene, and the gene has duplicated since moss [17]. Our data show that cpHsc70-1 is a group III precursor and cpHsc70-2 is a group II precursor (Figure 1C). We thus hypothesize that one of the reasons for transit peptide diversification after gene duplication is to achieve differential age-dependent import regulation through sequence differences in the transit peptides. To test our hypothesis, we found three more examples in which the protein is encoded by a single gene in *Chlamydomonas* but by a two-gene family in higher plants: chloroplast Cpn60α, Cpn10, and BCCP (sequences shown in Figure S7B–S7D). Import into chloroplasts of different ages showed that prCpn60α1 is a group I precursor and prCpn60α2 is a group II precursor, while prBCCP-1 is a group II precursor and prBCCP-2 is a group I precursor (Figure 7A). The prCpn10-1 protein has the initiation methionine as its single methionine, and thus the mature protein could not be observed after the removal of the transit peptide when the protein was labeled by [^35^S]Met. We thus added two methionine residues at its C terminus and created the protein prCpn10-1MM. Import of prCpn10-1MM and prCpn10-2 into chloroplasts of different ages showed that prCpn10-1MM is a group I precursor and prCpn10-2 is a group II precursor. We also tested a family in which the proteins are only present in higher plants, not in *Chlamydomonas*. The small J-domain containing protein DJC23 is encoded by a three-gene family, *DJC23*, *DJC24*, and *DJC66*, in *Arabidopsis*. DJC23 and DJC24 are more similar to each other than to DJC66 (Figure S7E for sequences). Import of the three precursor proteins into chloroplasts of different ages showed that prDJC23 and prDJC24 are group I precursors and prDJC66 is a group III precursor. However, not all gene family members fall into different age-selective groups. All three isoforms of *Arabidopsis* protochlorophyllide oxidoreductase are group III precursors (prPORA, prPORB, and prPORC; Figures 1C and S2).

If different isoforms fall into different age-selective groups because of sequence differences in their transit peptides, then changing the sequences should change the age-selective group of the precursor. One of the regions that is different between the transit peptides of cpHsc70-1 and cpHsc70-2 is around residues 23 and 24 of cpHsc70-1, where the two positively charged amino acids Lys23-Arg24 in cpHsc70-1 are replaced with Thr-Lys at the corresponding position in cpHsc70-2 (Figure 7B). Since we have shown that two consecutive positive charges is a necessary motif for group III transit peptides, we mutated Lys23 and Arg24 in cpHsc70-1 to Thr and Lys to resemble the sequence in cpHsc70-2 and created the mutant prcpHsc70-1(KR2324TK). Import of prcpHsc70-1(KR2324TK) into chloroplasts of different ages showed that it was changed to a group II precursor, just like prcpHsc70-2 (Figure 7B).

## Discussion

We have identified a new level of differential regulation residing at the stage of protein import into chloroplasts. We have further identified a necessary transit peptide motif specifying a preference for older chloroplasts. To our knowledge, this is the first functional motif identified for chloroplast-targeting transit peptides [18],[19]. Most likely specific motifs are also present for the other two age-selective groups. These motifs are actually one set of tools available to multicellular organisms for differential age-specific regulation.

The lack of differentially regulated import in *Chlamydomonas* suggests that differential regulation is not just used to select for proteins that are needed in young versus mature chloroplasts. Because of the limited number of precursors tested, it is difficult to generalize the function of proteins in each age-selective group. However, upright growth of higher plants results in the unique situation of having leaves of different ages on the same stem, exposed to different environmental and nutrient conditions. In addition, mature chloroplasts are maintained for a longer period of time. Group I proteins may be more important for chloroplast functions during rapid cell division and expansion under normal or high light conditions. Group II proteins may be the true housekeeping proteins that are needed at a similar level under all conditions, and Group III proteins, which in unicellular algae such as *Chlamydomonas* would be down-regulated like other chloroplast proteins in anticipation for the next cell division, may need to be maintained at a higher level in higher plants because they are important for long-term maintenance of chloroplasts under lower light conditions.

How the three groups of transit peptides are decoded by receptors on the chloroplast surface remains to be investigated. They may interact with the same receptor with different affinities, or each may interact with a specific receptor. In *Arabidopsis*, the major receptor Toc159 is encoded by a four-gene family that is separated into three subfamilies, Toc159, Toc132, and Toc90. Analyses of grape, rice [20], soybean, and *Medicago truncatula* (Figure S8) genomes indicate that their Toc159 families are also separated into the same three subgroups, suggesting that these three Toc159 subfamilies are most likely also present in pea. Based on small-scale analyses, different Toc159 subfamily members were shown to have different substrate preferences, and it was proposed that in *Arabidopsis*, the atToc159 (“at” for *A. thaliana*) subfamily functions as a selective receptor for photosynthetic proteins and the atToc132 subfamily functions as a selective receptor for housekeeping proteins [21]–[24]. However, recent large-scale proteomic and transcriptomic analyses of *attoc159* mutants revealed an unexpected client protein promiscuity of atToc159, indicating that the original separation of photosynthetic versus housekeeping is too simplistic [25]. Nonetheless, two transit peptides, those of prRBCS and of prFd, were shown directly to preferentially bind to atToc159 [22], and both precursors are group I precursors from our study. One transit peptide, that of prPDH, was shown directly to preferentially bind to atToc132 [24], a group II protein from our study. Proteomic analyses of the *attoc159* and *attoc132* mutants also support that Toc159 may be the receptor for group I precursors and Toc132 may be the receptor for group II precursors. Of the 44 atToc159-dependent proteins identified [25], only one, prRBCS, was tested by us, and it is indeed a group I precursor. Of the 308 Toc159-independent proteins identified, we have tested nine precursors, and eight are in group II or III. The only exception is prCpn10-1 (At2g44650), which is classified as an atToc159-independent protein but is a group I protein in our analyses. Cpn10 is encoded by two highly similar genes, *CPN10-1* and *CPN10-2*, and the two proteins share 79.4% identity. Cpn10-2 is a group II protein. It is possible that some of the peptides from these two proteins were not distinguished. Of the 19 proteins whose abundance was increased or not changed in the *attoc132* mutant [21], we and others [8] have tested five, and all five are group I or group III precursors, suggesting that atToc132 is less important for the import of group I and III precursors. However, the overlap between the proteins identified by the proteomic approaches and the proteins tested by us is small. More work is needed to identify the receptors for the three groups of precursors.

Our preliminary data suggest that the receptor for group III precursors is a thermolysin-sensitive chloroplast surface protein that is present in increasing amounts as chloroplasts age, but its identity remains to be uncovered. We also cannot exclude that some post-translational modifications that increase with age on one of the Toc159 family members are responsible for the increased import of group III precursors in older chloroplasts. The two consecutive positive charges in group III transit peptides may interact with some negatively charged modifications, like phosphorylations, on one of the receptors.

More and more evidence indicates that organellar protein import is specifically regulated. The biogenesis and functions of three mitochondrial outer-membrane translocon proteins have recently been shown to be affected by phosphorylations through two cytosolic kinases [26]. Furthermore, during mitochondrial stress, protein import into mitochondria is reduced, allowing the protein ATFS-1 (activating transcription factor associated with stress-1) to be imported into the nucleus, which is important for activation of the mitochondrial unfolded protein response [27]. In addition, the presence of a specific degradation system in the cytosol for chloroplast precursor proteins [28] also supports our finding that protein import into chloroplasts is regulated. In vitro, phosphorylation and exogenously added calmodulin inhibitor and redox reagents have also been shown to affect the function and association of some chloroplast translocon components [29]–[31]. Whether these modifications take place in vivo remains to be investigated.

It has long been puzzling why the signal peptides for protein targeting to ER, mitochondria, and chloroplasts are so diverse at the amino acid sequence level. It has been proposed for ER-targeting signal peptides that the heterogeneity in sequences has regulatory functions [5]. It has been shown that during acute ER stress, translocation of ER proteins is attenuated in a signal-peptide-selective manner [32]. In addition, the intrinsic inefficiency of the prion protein signal peptide is required for pathogenesis of some prion mutations. Therefore, different signal peptide efficiencies, resulting from differences in sequences, can have a significant impact on organism physiology [33]. Here we show that sequences of chloroplast transit peptides determine the age selectivity of precursor proteins. Furthermore, different members of a gene family often belong to different age-selective groups because of sequence differences in their transit peptides. Therefore, the sequence diversity of these transit peptides has evolved to mediate age-selective regulation. Multi-gene family members of ER and mitochondrial proteins also share the property that they display high sequence similarity in the mature protein region but have more diverse signal sequences. Thus, it is likely that diversity in signal peptide sequences among gene family members has evolved to mediate protein transport regulation in ER and mitochondria as well.

## Materials and Methods

### Plant Growth and Chloroplast Isolation

Pea (*Pisum sativum* cv. Little Marvel) seedlings were grown on vermiculite in growth chambers under a 12 h light/12 h dark cycle at 20°C. Chloroplasts were isolated from pea seedlings as described [34] except that a blender, instead of a Kinematica Polytron homogenizer, was used. The cell-wall-deficient strain of *C. reinhardtii*, *CC-400 cw15*, was inoculated in TAP medium [35] at a density of 4×10^4^ cells ml^−1^ and grown under continuous light. After 2 d, the cell cultures were switched to 12 h light/12 h dark cycles. Chloroplasts were isolated from cultures harvested 1, 6, and 11 h after the start of the third light cycle as described [36], except that a Dounce tissue grinder (Wheaton) was used to break the cells. *Arabidopsis* plants were grown on MS synthetic agar medium with 2% sucrose as described [11]. Chloroplast numbers were counted under an Olympus BH2 phase-contrast microscope with a hemocytometer.

### Import and Post-Import Treatments

All precursor proteins were translated in vitro by the TNT-wheat germ or reticulocyte lysate system (Promega) in the presence of [^35^S]Met. A typical import reaction with pea chloroplasts was performed in a 60-µl reaction containing 20–25 µl of in vitro–translated precursor proteins, chloroplasts (20 µg of chlorophylls, see below), 3 mM Mg-ATP in 1× import buffer (330 mM sorbitol, 50 mM HEPES-KOH [pH 8.0]) for 25 min (see below) at room temperature. For most precursors, prHsp93 was co-imported for quantification normalization. For some precursors, because of the position of their mature protein or their lower import efficiency, signals from hemoglobin from the reticulocyte lysates or the imported Hsp93 degradation fragments may interfere with quantification, and endogenous cpHsc70 was analyzed by immunoblotting for confirmation and/or normalization. Competition experiments using recombinant prRBCS were performed as described [37],[38]. In brief, import was performed with [^35^S]Met-labeled precursors and various concentrations of recombinant prRBCS at room temperature for 15 min. All reactions had the same concentration of urea as the reaction with the highest amount of recombinant proteins. After import, an excess amount of ice-cold 40% Percoll in 1× import buffer was added to stop the import reaction, and intact chloroplasts were pelleted and washed. Where indicated, chloroplasts were further treated with 200 µg ml^−1^ thermolysin as described [34]. Import into *Chlamydomonas* chloroplasts was performed as described [16]. In brief, [^35^S]Met-labeled precursors were incubated with isolated chloroplasts in the presence of 10 mM Mg-ATP in import buffer (250 mM sorbitol, 1.5 mM MgCl_2_, 1 mM MnCl_2_, 0.1 mM Na_2_HPO_4_, 2 mM Na_2_-EDTA, 35 mM HEPES-KOH [pH 7.8]) for 20 min at room temperature. For the import of prTic40 and prOE23 into *Chlamydomonas* chloroplasts, we found that import efficiencies were much higher when import was performed in the import buffer for pea chloroplasts (330 mM sorbitol, 50 mM HEPES-KOH [pH 8.0]) containing 5 mM Mg-ATP, and still showed the same pattern of developmental regulation. Thus, for these two precursors data obtained using the pea chloroplast import buffer are presented.

Chloroplasts of different ages have several different characteristics. For example, older chloroplasts have more chlorophylls per chloroplast and are also larger in size and have larger surface area [8],[39] and possibly more receptors per chloroplast. We therefore used two methods for normalization during import. Import reactions were performed with either an equal number of chloroplasts or an equal amount of chlorophyll in each reaction. The three groups of age-selective import patterns were obtained with both methods (Figure S3A). However, we found that chlorophyll measurements were more accurate and consistent, and significant variations were often obtained with chloroplast number counting from different counts and dilutions. We therefore used an equal amount of chlorophyll per reaction for further characterizations. The same age-selective import patterns were also observed when import was performed for 10 min (Figure S3B).

Protein samples were analyzed by SDS-PAGE using the NuPAGE gel system (Invitrogen), exposed to X-ray films, and quantified using a phosphorimager (Fuji FLA-5000 imaging system). Some samples were additionally analyzed by immunoblotting as described [40]. Immunoblots were quantified using the Luminescent Image Analyzer LAS1000 Plus and Image Gauge version 4.0 software (Fujifilm). The antibodies against glutamine synthetase 2 (AS08 296), CAB (AS01 004), and *Chlamydomonas* chloroplast stromal Hsp70B (AS06 175) were purchased from Agrisera.

The antibody against mitochondrial porin was produced by cloning the gene encoding *Arabidopsis* mitochondrial porin (At3g01280) into the pGEX-5X-1 vector. The GST-porin fusion protein was purified from *E. coli* and used as antigen for immunization in rabbits. The antibody was used at a dilution of 1∶1,000. Antibodies against Toc159 [38], Tic110 [38], Toc75 [38], Tic40 [11], and cpHsc70/S78 [41] were produced and used as described.

### *Arabidopsis* Transformation and Chloroplast Fractionation

The *TIC40p:prTic40* and *TIC40p:RBCStp-mTic40* transgenes were cloned into the binary vector pPZP221 [42], introduced into *Agrobacterium tumefaciens* GV3101 and transformed into the *tic40-1* mutant [11] using the floral spray method [43]. Independent transgenic lines containing at least one copy of the transgene were selected from subsequent generations and used for further analyses. *Arabidopsis* chloroplast isolation and fractionation were performed as described [44].

### DNA Clones and Fusion and Mutant Constructs

The full names of precursors used in this study, and their accession numbers and species, are listed in Table S1. EST clones of *C. reinhardtii* Cr-prRBCS and Cr-prL11 were obtained from Kazusa DNA Research Institute [45]. Primers used for building fusion constructs are listed in Table S2. For the construction of RBCStp-mTic40, the RBCStp-mTic40 fragment was amplified by two-step PCR. The first rounds of PCR were performed to generate the soybean RBCS transit peptide (RBCStp) from prRBCS [46] and the Tic40 mature region from an *Arabidopsis* Tic40 cDNA that was subcloned into the pSP72 plasmid (pSP72-atTic40). The RBCStp-mTic40 fragment was then amplified by a second round of PCR using the two PCR products from the first rounds as the template and with a forward primer adding a BamHI site and a T7 primer as the reverse primer. The fragment was cloned into the BamHI/EcoRI site of pSP72, creating the plasmid pSP72-RBCStp-mTic40. For Tic40tp-mRBCS, the Tic40 transit peptide (Tic40tp) fragment was amplified by PCR from pSP72-atTic40, with a forward primer adding an XhoI site and a reverse primer adding an SphI site. The amplified fragment was subcloned into the XhoI/SphI site of the plasmid pSP72-mSS [47]. For RBCStp-GST, the pea prRBCS transit peptide fragment was amplified by PCR from a plasmid containing the pea prRBCS cDNA cloned into the pSP64 plasmid, with the forward and reverse primers both adding an EcoNI site to the PCR fragment. The fragment was digested with EcoNI and subcloned into the EcoNI site of the vector pGEX-5X-1 [48]. The RBCStp-GST fragment was then amplified by PCR, with a forward primer adding a BamHI site and a reserve primer adding an EcoRI site, and the amplified fragment was subcloned into the BamHI/EcoRI site of pSP72, resulting in the plasmid pSP72-PsRBCStp-GST. The soybean prRBCS transit peptide was excised from the soybean–pea hybrid prRBCS [46] using HindIII and SphI and cloned into the HindIII/SphI site of pSP72-PsRBCStp-GST to replace the pea prRBCS transit peptide. The resulting plasmid was called pSP72-RBCStp-GST and was used to produce RBCStp-GST for import assays. For Tic40tp-GST, the Tic40 transit peptide fragment was amplified by PCR from a plasmid containing the pea prTic40 cDNA cloned into pBluescript, with a T7 primer and a reverse primer adding an SphI site. The fragment was then subcloned into the XhoI/SphI site of pSP72-PsRBCStp-GST, replacing the pea prRBCS transit peptide. For *TIC40p:prTic40* and *TIC40p:RBCStp-mTic40*, the two fragments were amplified by two-step PCR. The first rounds of PCR were performed to generate the *TIC40* 2-kb promoter fragment from *Arabidopsis* leaf genomic DNA and the Tic40 full-length cDNA from pSP72-atTic40, or the RBCStp-mTic40-encoding fragment from pSP72-RBCStp-mTic40. The second rounds of PCR were performed to generate the *TIC40p:prTic40* and *TIC40p:RBCStp-mTic40* fragments using the first rounds of PCR products as templates and with a forward primer adding a KpnI site and a reserve primer adding an XbaI site to the PCR fragment. The amplified fragments were subcloned into the KpnI/XbaI site of the transformation vector pPZP221 [42].

For the nine-amino-acid alanine block scanning mutations of prTic40 transit peptide, a PCR approach was used to introduce mutations into the transit peptides [49]. For each mutant, one pair of complementary primers was designed. The primers consisted of mutated residues in the central region, flanked by wild-type sequences. With the wild-type template DNA and these primers, including the complementary pair, N-terminal, and C-terminal primers, the first round of PCR was performed to generate two fragments, the 5′ and 3′ segments. The second round of PCR was performed with the 5′ and 3′ segments as templates, and the N-terminal and C-terminal primers. The PCR products were subcloned and then sequenced. For amino acid changes, the QuikChange Site-Directed Mutagenesis Kit was used (Agilent Technologies).

## Supporting Information

Figure S1 **The protein levels of pea chloroplast cpHsc70 and** ***Chlamydomonas*** **chloroplast Hsp70B remain constant during the developmental stages analyzed.** (A) Chloroplasts were isolated from pea leaves of different ages, and total chloroplast proteins were analyzed by SDS-PAGE followed by Coomassie Blue staining, or immunoblotting using an antibody against cpHsc70. An equal number of chloroplasts were loaded in each lane. (B) Chloroplasts were isolated from synchronized cultures of *Chlamydomonas* 1, 6, and 11 h after the start of the third light cycle, and total chloroplast proteins were analyzed by SDS-PAGE followed by Coomassie Blue staining, or immunoblotting using the antibody against Hsp70B. An equal number of chloroplasts were loaded in each lane. The positions of endogenous RBCL, CAB, and RBCS in pea chloroplasts are labeled.
(PDF)

Figure S2 **Test of age selectivity of more chloroplast precursor proteins.** Various precursor proteins were imported into chloroplasts isolated from pea leaves of different ages. Intact chloroplasts were re-isolated after the import reaction and analyzed by SDS-PAGE and autoradiography. The amount of endogenous cpHsc70 or co-imported Hsp93 of the same sample was analyzed and used for normalization in quantifications shown in the bar graph below each gel. The amount of mature proteins imported in leaf 1 was set as 100%. Data shown are means ± SD, *n* = 3. m, mature form; p, precursor form; TR, in vitro–translated precursor proteins before import.
(PDF)

Figure S3 **Three age-selective groups were observed using different import conditions.** (A) Import of prRBCS, prHsp93, and prTic40 using an equal number of chloroplasts in each reaction. Chloroplasts were isolated from leaves of different ages and used for import experiments. Each reaction contained 25 µl of in vitro–translated precursor proteins, 1.5×10^6^ chloroplasts, 3 mM Mg-ATP, and import buffer to a final volume of 60 µl. Import was performed for 25 min at room temperature. The TR lane for the prTic40 panel is from a longer exposure of the same gel of the import samples. A representative result of three independent experiments is shown. (B) Ten-minute import reactions of the same three representative precursors. All three precursors were imported together in the same reaction. Each reaction contained 10 µl of prRBCS, 5 µl of prHsp93, 10 µl of prTic40, 20 µg of chlorophylls of chloroplasts, 3 mM Mg-ATP, and import buffer to a final volume of 60 µl. Import was performed for 10 min at room temperature. A representative result of two independent experiments is shown. For both (A) and (B), intact chloroplasts were re-isolated and analyzed by SDS-PAGE and autoradiography. Equal numbers of chloroplasts were loaded in each lane. The amount of cpHsc70 in each sample was analyzed by immunoblotting. m, mature form; p, precursor form; TR, in vitro–translated precursor proteins before import.
(PDF)

Figure S4 **Analyses of Tic40 proteins in the** ***TIC40*** **transgenic plants.** (A) All *TIC40* transgenic plants have a comparable steady state Tic40 protein level. Total protein extracts of plants shown in Figure 4B were analyzed by immunoblotting. Mitochondrial porin and chloroplast Tic110 and Toc75 were analyzed as controls. The relative amount of Tic40, normalized to the amount of porin in the same sample and with the level in the wild type set as 1, is labeled beneath the Tic40 blot. (B) Tic40 proteins were localized in the inner envelope membrane. Chloroplasts were isolated from the #2 line of the *TIC40p:RBCStp-mTic40* transgenic plants and the #2 line of the *TIC40p:prTic40* transgenic plants and fractionated as described [44]. The localizations of Toc75 (outer envelope membrane, OM), Tic110 (inner envelope membrane, IM), glutamine synthetase 2 (GS2; stroma, S), and CAB (thylakoid, Thy.) in the same samples were also analyzed as markers for each fraction. T, total chloroplasts.
(PDF)

Figure S5 **Import of Cr-prRBCS and Cr-prL11 into pea chloroplasts of different ages.** Cr-prRBCS is recognized as a group I precursor, and Cr-prL11 is recognized as a group III precursor by pea chloroplasts. Precursor proteins were imported into chloroplasts isolated from pea leaves of different ages. Intact chloroplasts were re-isolated after the import reaction and analyzed by SDS-PAGE and autoradiography. Cr-prL11 was processed into multiple bands after import into pea chloroplasts. Thermolysin treatments of chloroplasts after import were used to confirm that all the bands quantified were within chloroplasts (data not shown). The amount of mature Hsp93 imported in the same sample was used for normalization in quantifications shown in the bar graph below each gel. The amount of mature proteins imported in leaf 1 was set as 100%. Data shown are means ± SD, *n* = 3. m, mature form; p, precursor form; TR, in vitro–translated precursor proteins before import.
(PDF)

Figure S6 **Import of prTic40 and prL11 transit peptide mutants.** (A) Post-import trypsin treatment of prTic40 and prTic40-A(10–18). prHsp93 was co-imported with prTic40 or prTic40-A(10–18). Chloroplasts after import were treated with trypsin. For the post-import trypsin treatment, the import assay was stopped by adding an excess amount of ice-cold import buffer, separated into two halves and spun down at 3,800 rpm for 5 min. One half was resuspended in import buffer, and the other half was resuspended in import buffer containing 100 µg ml^−1^ trypsin. Both reactions were incubated in the dark at room temperature for 1 h. Trypsin digestion was stopped by adding one-tenth volume of 20 mg ml^−1^ trypsin inhibitor. The reaction mixture was kept in the dark on ice for 10 min. Intact chloroplasts were re-isolated. All samples were analyzed by SDS-PAGE and autoradiography. Samples were also analyzed by SDS-PAGE followed by immunoblotting analyses of Toc75, which is trypsin sensitive, to confirm the effectiveness of the trypsin treatment. O, chloroplasts from leaf 4; TR, in vitro–translated precursor protein; Y, chloroplasts from leaf 1. (B) The prL11(KK4445EE) mutation specifically reduced import into old chloroplasts. Import efficiency was calculated as the percentage of precursor proteins added to the import reaction that was imported into the chloroplasts. Each value was then normalized to the value of the wild-type imports into chloroplasts of the same age.
(PDF)

Figure S7 **Sequence alignment of gene family isoforms analyzed.** (A) cpHsc70 family; (B) BCCP family; (C) Cpn60 family; (D) Cpn10 family; (E) DJC23 family. Transit peptide processing sites, as predicted by ChloroP, are indicated by arrows.
(PDF)

Figure S8 **Toc159 family members in soybean (** ***Glycine max*** **) and** ***M. truncatula*** **are also separated into three subgroups.** Soybean and *M. truncatula* Toc159 homolog sequences were retrieved from PlantGDB (http://www.plantgdb.org). The prefix “Gm” is used for soybean, “Mt” for *M. truncatula*, and “At” for *Arabidopsis* sequences. Multiple sequence alignment was performed using the BLOSUM protein weight matrix, and the phylogenetic tree was constructed using the neighbor-joining method of the ClustalX program. The unrooted neighbor-joining tree was visualized by the TreeView program (http://taxonomy.zoology.gla.ac.uk/rod/treeview.html). Bootstrap values shown on the branches were computed with 1,000 replicates. Three clades were supported by 100% of the bootstrap value.
(PDF)

Table S1 **Full names and accession numbers of precursors used in this study.** Transit peptide sequences of group III precursors are also included. Two consecutive amino acids with positive charges are underlined. Transit peptide processing site is based on ChloroP prediction or published literature. Two consecutive positive charges are not found within the ChloroP-predicted transit peptide of DJC66 (predicted processing site indicated by an upward arrow), but three consecutive Arg are found eight amino acids downstream. Proteins with accession numbers with “Atxg” are from *Arabidopsis*.
(DOC)

Table S2 **Primers used for building fusion constructs.** The restriction enzyme sites used for cloning are underlined.
(DOC)

## Abbreviations

CAB: light-harvesting complex of photosystem II
cpHsc70: chloroplast stromal Hsp70
ER: endoplasmic reticulum
RBCL: the large subunit of RuBP carboxylase
RBCS: small subunit of RuBP carboxylase
SD: standard deviation
Toc: translocon at the outer-envelope-membrane of chloroplasts
Tic: translocon at the inner-envelope-membrane of chloroplasts
