# Supplementary figures and images for: Differential Age-Dependent Import Regulation by Signal Peptides
Source: PLoS Biol. 2012 Oct 30;10(10):e1001416. doi: 10.1371/journal.pbio.1001416 (PMC3484058; doi:10.1371/journal.pbio.1001416)

Supplementary Information Figure S1

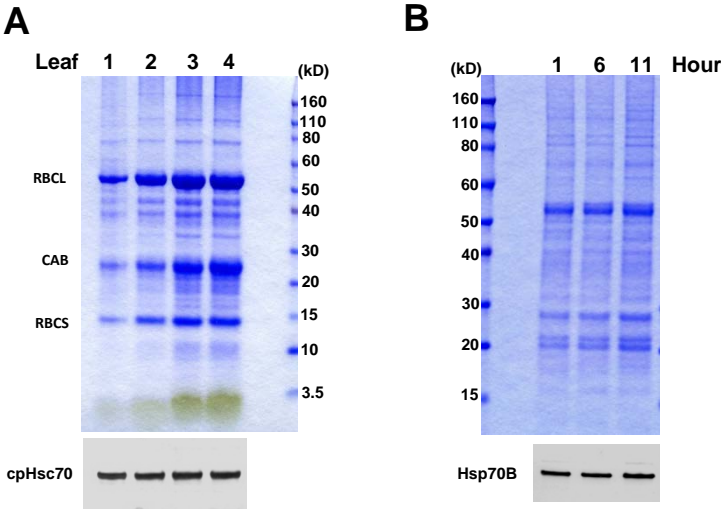

Supplement: Figure S1 — The protein levels of pea chloroplast cpHsc70 and Chlamydomonas chloroplast Hsp70B remain constant during the developmental stages analyzed. (A) Chloroplasts were isolated from pea leaves of different ages, and total chloroplast proteins were analyzed by SDS-PAGE followed by Coomassie Blue staining, or immunoblotting using an antibody against cpHsc70. An equal number of chloroplasts were loaded in each lane. (B) Chloroplasts were isolated from synchronized cultures of Chlamydomonas 1, 6, and 11 h after the start of the third light cycle, and total chloroplast proteins were analyzed by SDS-PAGE followed by Coomassie Blue staining, or immunoblotting using the antibody against Hsp70B. An equal number of chloroplasts were loaded in each lane. The positions of endogenous RBCL, CAB, and RBCS in pea chloroplasts are labeled. (PDF) [file pbio.1001416.s001.pdf]

Supplementary Information Figure S2

Group I

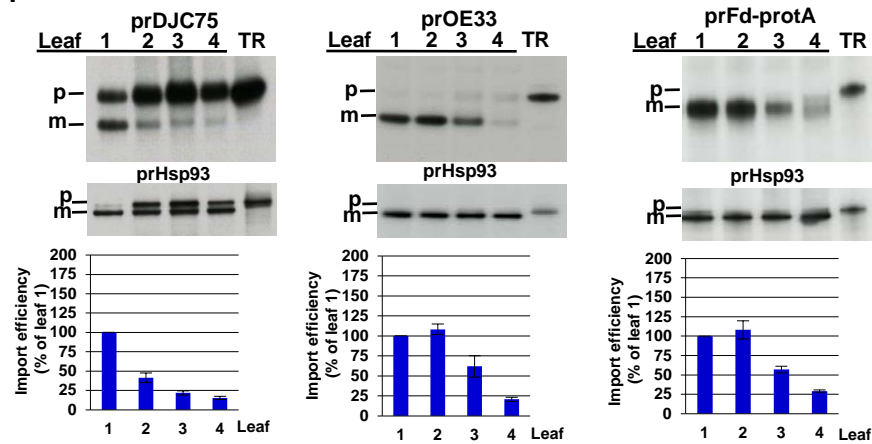

Group III

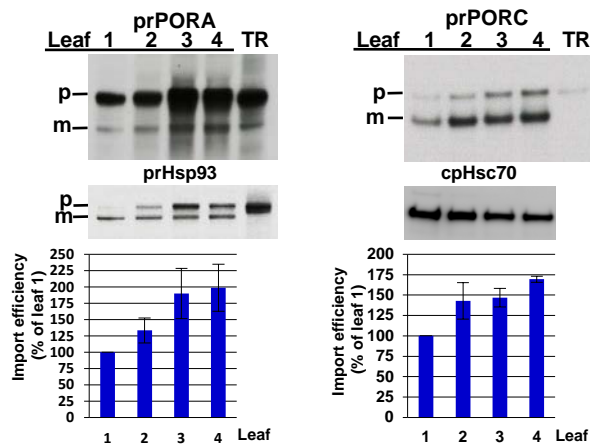

Supplement: Figure S2 — Test of age selectivity of more chloroplast precursor proteins. Various precursor proteins were imported into chloroplasts isolated from pea leaves of different ages. Intact chloroplasts were re-isolated after the import reaction and analyzed by SDS-PAGE and autoradiography. The amount of endogenous cpHsc70 or co-imported Hsp93 of the same sample was analyzed and used for normalization in quantifications shown in the bar graph below each gel. The amount of mature proteins imported in leaf 1 was set as 100%. Data shown are means ± SD, n = 3. m, mature form; p, precursor form; TR, in vitro–translated precursor proteins before import. (PDF) [file pbio.1001416.s002.pdf]

Supplementary Information Figure S3

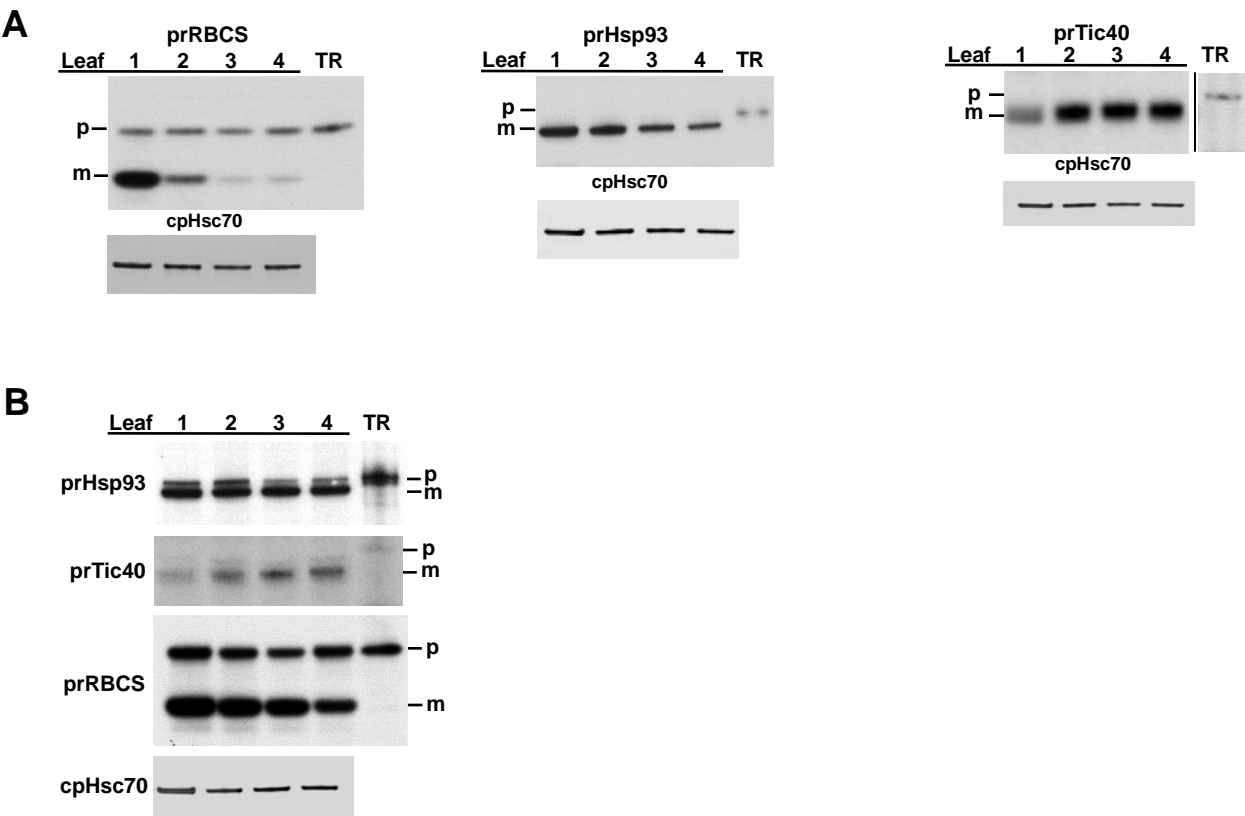

Supplement: Figure S3 — Three age-selective groups were observed using different import conditions. (A) Import of prRBCS, prHsp93, and prTic40 using an equal number of chloroplasts in each reaction. Chloroplasts were isolated from leaves of different ages and used for import experiments. Each reaction contained 25 µl of in vitro–translated precursor proteins, 1.5×10^6^ chloroplasts, 3 mM Mg-ATP, and import buffer to a final volume of 60 µl. Import was performed for 25 min at room temperature. The TR lane for the prTic40 panel is from a longer exposure of the same gel of the import samples. A representative result of three independent experiments is shown. (B) Ten-minute import reactions of the same three representative precursors. All three precursors were imported together in the same reaction. Each reaction contained 10 µl of prRBCS, 5 µl of prHsp93, 10 µl of prTic40, 20 µg of chlorophylls of chloroplasts, 3 mM Mg-ATP, and import buffer to a final volume of 60 µl. Import was performed for 10 min at room temperature. A representative result of two independent experiments is shown. For both (A) and (B), intact chloroplasts were re-isolated and analyzed by SDS-PAGE and autoradiography. Equal numbers of chloroplasts were loaded in each lane. The amount of cpHsc70 in each sample was analyzed by immunoblotting. m, mature form; p, precursor form; TR, in vitro–translated precursor proteins before import. (PDF) [file pbio.1001416.s003.pdf]

Supplementary Information Figure S4

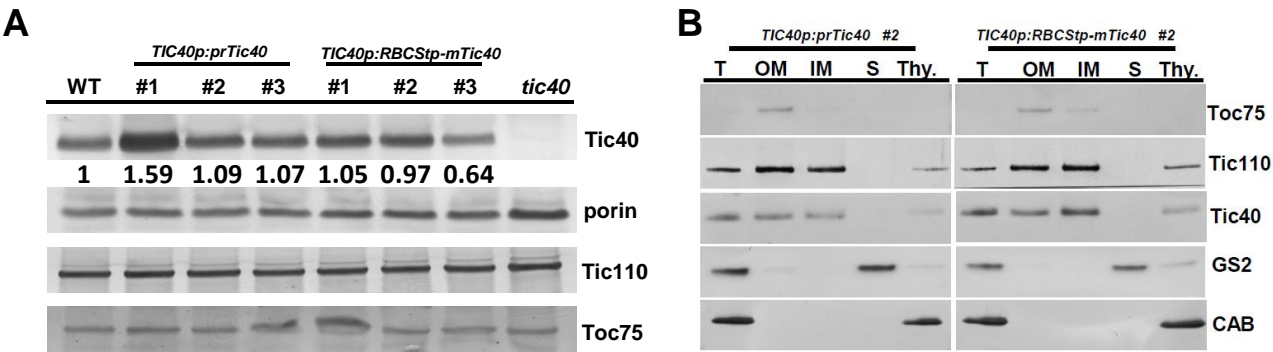

Supplement: Figure S4 — Analyses of Tic40 proteins in the TIC40 transgenic plants. (A) All TIC40 transgenic plants have a comparable steady state Tic40 protein level. Total protein extracts of plants shown in Figure 4B were analyzed by immunoblotting. Mitochondrial porin and chloroplast Tic110 and Toc75 were analyzed as controls. The relative amount of Tic40, normalized to the amount of porin in the same sample and with the level in the wild type set as 1, is labeled beneath the Tic40 blot. (B) Tic40 proteins were localized in the inner envelope membrane. Chloroplasts were isolated from the #2 line of the TIC40p:RBCStp-mTic40 transgenic plants and the #2 line of the TIC40p:prTic40 transgenic plants and fractionated as described [44]. The localizations of Toc75 (outer envelope membrane, OM), Tic110 (inner envelope membrane, IM), glutamine synthetase 2 (GS2; stroma, S), and CAB (thylakoid, Thy.) in the same samples were also analyzed as markers for each fraction. T, total chloroplasts. (PDF) [file pbio.1001416.s004.pdf]

Supplementary Information Figure S5

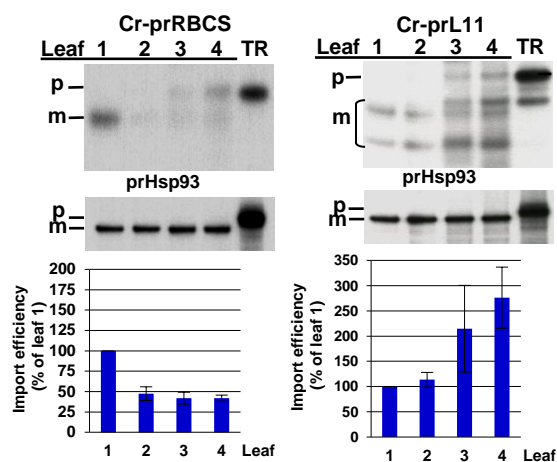

Supplement: Figure S5 — Import of Cr-prRBCS and Cr-prL11 into pea chloroplasts of different ages. Cr-prRBCS is recognized as a group I precursor, and Cr-prL11 is recognized as a group III precursor by pea chloroplasts. Precursor proteins were imported into chloroplasts isolated from pea leaves of different ages. Intact chloroplasts were re-isolated after the import reaction and analyzed by SDS-PAGE and autoradiography. Cr-prL11 was processed into multiple bands after import into pea chloroplasts. Thermolysin treatments of chloroplasts after import were used to confirm that all the bands quantified were within chloroplasts (data not shown). The amount of mature Hsp93 imported in the same sample was used for normalization in quantifications shown in the bar graph below each gel. The amount of mature proteins imported in leaf 1 was set as 100%. Data shown are means ± SD, n = 3. m, mature form; p, precursor form; TR, in vitro–translated precursor proteins before import. (PDF) [file pbio.1001416.s005.pdf]

Supplementary Information Figure S6

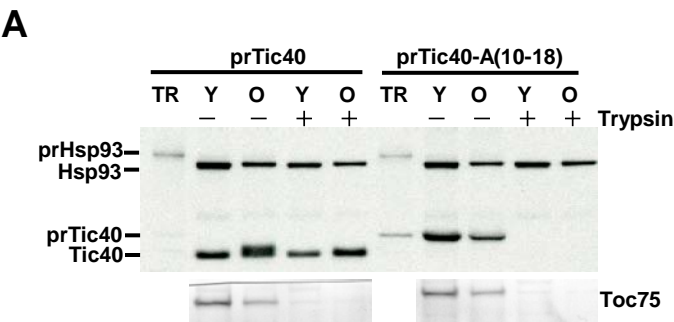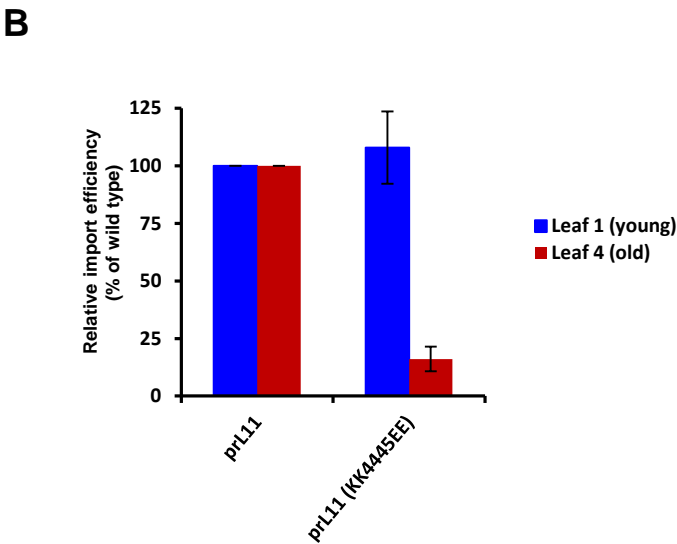

Supplement: Figure S6 — Import of prTic40 and prL11 transit peptide mutants. (A) Post-import trypsin treatment of prTic40 and prTic40-A(10–18). prHsp93 was co-imported with prTic40 or prTic40-A(10–18). Chloroplasts after import were treated with trypsin. For the post-import trypsin treatment, the import assay was stopped by adding an excess amount of ice-cold import buffer, separated into two halves and spun down at 3,800 rpm for 5 min. One half was resuspended in import buffer, and the other half was resuspended in import buffer containing 100 µg ml−1 trypsin. Both reactions were incubated in the dark at room temperature for 1 h. Trypsin digestion was stopped by adding one-tenth volume of 20 mg ml−1 trypsin inhibitor. The reaction mixture was kept in the dark on ice for 10 min. Intact chloroplasts were re-isolated. All samples were analyzed by SDS-PAGE and autoradiography. Samples were also analyzed by SDS-PAGE followed by immunoblotting analyses of Toc75, which is trypsin sensitive, to confirm the effectiveness of the trypsin treatment. O, chloroplasts from leaf 4; TR, in vitro–translated precursor protein; Y, chloroplasts from leaf 1. (B) The prL11(KK4445EE) mutation specifically reduced import into old chloroplasts. Import efficiency was calculated as the percentage of precursor proteins added to the import reaction that was imported into the chloroplasts. Each value was then normalized to the value of the wild-type imports into chloroplasts of the same age. (PDF) [file pbio.1001416.s006.pdf]

Supplementary Information Figure S8

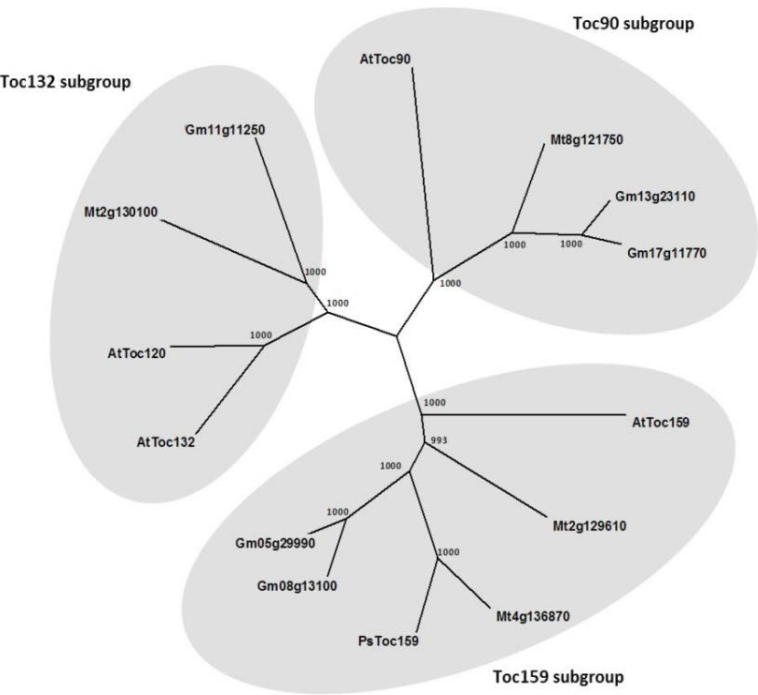

Supplement: Figure S8 — Toc159 family members in soybean ( Glycine max ) and M. truncatula are also separated into three subgroups. Soybean and M. truncatula Toc159 homolog sequences were retrieved from PlantGDB (http://www.plantgdb.org). The prefix “Gm” is used for soybean, “Mt” for M. truncatula, and “At” for Arabidopsis sequences. Multiple sequence alignment was performed using the BLOSUM protein weight matrix, and the phylogenetic tree was constructed using the neighbor-joining method of the ClustalX program. The unrooted neighbor-joining tree was visualized by the TreeView program (http://taxonomy.zoology.gla.ac.uk/rod/treeview.html). Bootstrap values shown on the branches were computed with 1,000 replicates. Three clades were supported by 100% of the bootstrap value. (PDF) [file pbio.1001416.s008.pdf]
