# Supplementary material for: Differential Age-Dependent Import Regulation by Signal Peptides
Source: PLoS Biol. 2012 Oct 30;10(10):e1001416. doi: 10.1371/journal.pbio.1001416 (PMC3484058; doi:10.1371/journal.pbio.1001416)
Supplement: Figure S7 — Sequence alignment of gene family isoforms analyzed. (A) cpHsc70 family; (B) BCCP family; (C) Cpn60 family; (D) Cpn10 family; (E) DJC23 family. Transit peptide processing sites, as predicted by ChloroP, are indicated by arrows. (PDF) [file pbio.1001416.s007.pdf]

Supplementary Information Figure S7

A

```
                                                                                 Section 1
                      (1) 1        10        20        30        40        50        60       79
cpHsc70-1 At4g24280   (1) MASSAAQIHVLGGIGFASSSSSK--RNLNGKGGTFMPRSAFFGTRTGPFSTPTSAFLRMGTRNGGGASRYAVGPVRVVN
cpHsc70-2 At5g49910   (1) MASSAAQIHILGGIGFPTSSSSSSTKNLDNKT-NSIPRSVFFGNRTSPFTTPTSAFLRMGRRNNN-ASRYTVGPVRVVN
                                                                                 Section 2
                     (80) 80       90        100       110       120       130       140     158
cpHsc70-1 At4g24280  (78) EKVVGIDLGTTNSAVAAMEGGKPTIVTNAEGQRTTPSVVAYTKSGDRLVGQIAKRQAVVNPENTFFSVKRFIGRKMNEV
cpHsc70-2 At5g49910  (78) EKVVGIDLGTTNSAVAAMEGGKPTIVTNAEGQRTTPSVVAYTKSKDRLVGQIAKRQAVVNPENTFFSVKRFIGRRMNEV
                                                                                 Section 3
                    (159) 159        170       180       190       200       210       220     237
cpHsc70-1 At4g24280 (157) DEESKQVSYRVVRDENNNVKLECPAINKQFAAEEISAQVLRKLVDDASRFLNDKVTKAVITVPAYFNDSQRTATKDAGR
cpHsc70-2 At5g49910 (157) AEESKQVSYRVIKDENGNVKLDCPAIGKQFAAEEISAQVLRKLVDDASRFLNDKVTKAVITVPAYFNDSQRTATKDAGR
                                                                                 Section 4
                    (238) 238         250       260       270       280       290       300     316
cpHsc70-1 At4g24280 (236) IAGLEVLRIINEPTAASLAYGFDRKANETILVFDLGGGTFDVSVLEVGDGVFEVLSTSGDTHLGGDDFDKRVVDWLAAE
cpHsc70-2 At5g49910 (236) IAGLEVLRIINEPTAASLAYGFERKSNETILVFDLGGGTFDVSVLEVGDGVFEVLSTSGDTHLGGDDFDKRVVDWLAST
                                                                                 Section 5
                    (317) 317          330       340       350       360       370       380     395
cpHsc70-1 At4g24280 (315) FKKDEGIDLLKDKQALQRLTEAAEKAKIELSSLTQTNMSLPFITATADGPKHIETTLTRAKFEELCSDLLDRVRTPVEN
cpHsc70-2 At5g49910 (315) FKKDEGIDLLKDKQALQRLTEAAEKAKIELSSLTQTNMSLPFITATADGPKHIETTLTRGKFEELCSDLLDRVRTPVEN
                                                                                 Section 6
                    (396) 396           410       420       430       440       450       460     474
cpHsc70-1 At4g24280 (394) SLRDAKLSFKDIDEVILVGGSTRIPAVQELVRKVTGKEPNVTVNPDEVVALGAAVQAGVLAGDVSDIVLLDVTPLSIGL
cpHsc70-2 At5g49910 (394) SLRDAKLSFKDIDEVILVGGSTRIPAVQDLVRKLTGKEPNVSVNPDEVVALGAAVQAGVLSGDVSDIVLLDVTPLSLGL
                                                                                 Section 7
                    (475) 475  480       490       500       510       520       530       540     553
cpHsc70-1 At4g24280 (473) ETLGGVMTKIIPRNTTLPTSKSEVFSTAADGQTSVEINVLQGEREFVRDNKSLGSFRLDGIPPAPRGVPQIEVKFDIDA
cpHsc70-2 At5g49910 (473) ETLGGVMTKIIPRNTTLPTSKSEVFSTAADGQTSVEINVLQGEREFVRDNKSIGSFRLDGIPPAPRGVPQIEVKFDIDA
                                                                                 Section 8
                    (554) 554    560       570       580       590       600       610       620     632
cpHsc70-1 At4g24280 (552) NGILSVSAVDKGTGKKQDITITGASTLPKDEVDQMVQEAERFAKDDKEKRDAIDTKNQADSVVYQTEKQLKELGEKIPG
cpHsc70-2 At5g49910 (552) NGILSVSASDKGTGKKQDITITGASTLPKDEVDTMVQEAERFAKEDKEKRDAIDTKNQADSVVYQTEKQLKELGEKIPG
                                                                                 Section 9
                    (633) 633    640       650       660       670       680       690       700     711
cpHsc70-1 At4g24280 (631) EVKEKVEAKLQELKDKIGSGSTQEIKDAMAALNQEVMQIGQSLYNQPGAGGPGAGPSPGGEGASSGDSSSSKGG--DGD
cpHsc70-2 At5g49910 (631) PVKEKVEAKLQELKEKIASGSTQEIKDTMAALNQEVMQIGQSLYNQPQPGG--ADSPPGGEASSSSDTSSSAKGGDNGG
                                                                                 Section 10
                    (712) 712      722
cpHsc70-1 At4g24280 (708) DVIDADFTDSQ
cpHsc70-2 At5g49910 (708) DVIDADFTDSN
```

B

```
                                                                                   Section 1
                    (1) 1        10        20        30        40        50        60        70        81
BCCP-1 At5g16390    (1) MASSSFSVTSPAAAASVYAVTQTSSHFPIQNRSRRVSFRLSAKPKLRFLSKPSRSSYPVVKAQSNKVSTGASSNAAKVDGP
BCCP-2 At5g15530    (1) MASLSVPCVKICALNRRVGSLPGISTQRWQPQPNGISFPSDVSQNHSAFWRLRATTNEVVSNSTPMTNGGYMNGKAKTNVP
                                                                                   Section 2
                   (82) 82      90        100       110       120       130       140       150      162
BCCP-1 At5g16390   (82) SSAEGKEKNSLKESSASSPELATEESISEFLTQVTTLVKLVDSRDIVELQLKQLDCELVIRKKEALPQPQAPASYVMMQQP
BCCP-2 At5g15530   (82) EPAE----------------------LSEFMAKVSGLLKLVDSKDIVELELKQLDCEIVIRKKEALQQAVPPAPVYHSMPP
                                                                                   Section 3
                  (163) 163    170       180       190       200       210       220       230        243
BCCP-1 At5g16390  (163) NQPSYAQQMAPPAAPAAAAPAPSTPASLPPPSPPTPAKSSLPTVKSPMAGTFYRSPAPGEPPFIKVGDKVQKGQVLCIVEA
BCCP-2 At5g15530  (141) VMADFSMPPAQPVALPPSPTPTSTPATAKPTSAPS---SSHPPLKSPMAGTFYRSPGPGEPPFVKVGDKVQKGQIVCIIEA
                                                                                   Section 4
                  (244) 244   250       260       270      280
BCCP-1 At5g16390  (244) MKLMNEIESDHTGTVVDIVAEDGKPVSLDTPLFVVQP
BCCP-2 At5g15530  (219) MKLMNEIEAEKSGTIMELLAEDGKPVSVDTPLFVIAP
```

C

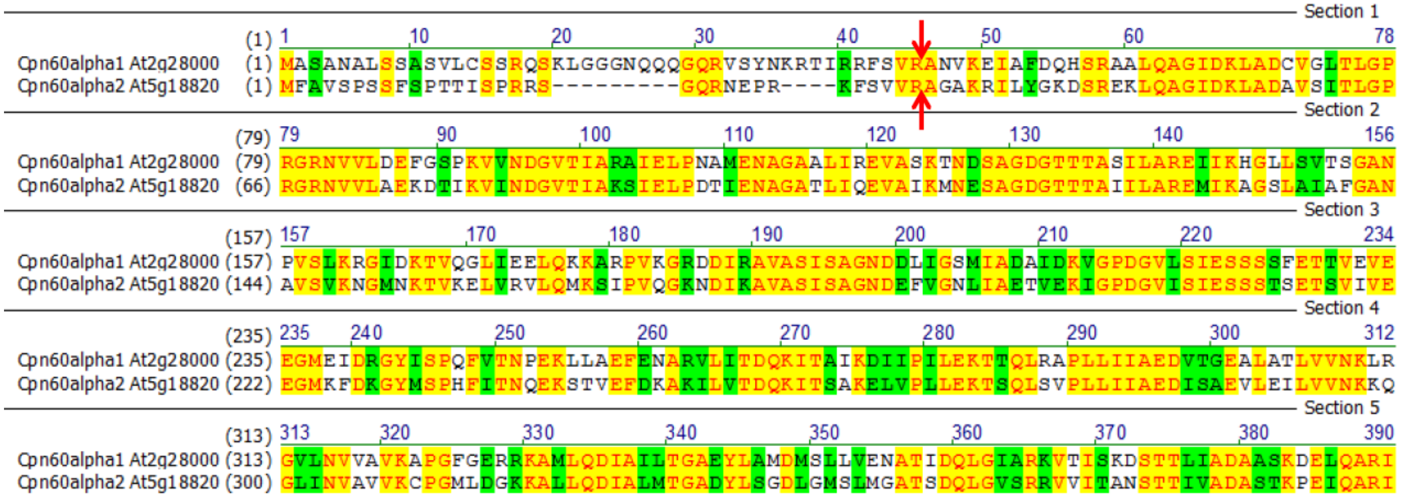
Section 1
(1) 1 10 20 30 40 50 60 78
Cpn60alpha1 At2g28000 (1) MASANALSSASVLCSSRQSKLGGGNQQQGQRVSYNKRTIRRFSVRANVKEIAFDQHSRAALQAGIDKLADCVGLTLGP
Cpn60alpha2 At5g18820 (1) MFAVSPSSFSPTTISPRRS---------GQRNEPR----KFSVVRAGAKRILYGKDSREKLQAGIDKLADAVSITLGP
Section 2
(79) 79 90 100 110 120 130 140 156
Cpn60alpha1 At2g28000 (79) RGRNVVLDEFGSPKVVNDGVTIARAIELPNAMENAGAALIREVASKTNDSAGDGTTTASILAREIIKHGLLSVTSGAN
Cpn60alpha2 At5g18820 (66) RGRNVVLAEKDTIKVINDGVTIAKSIELPDTIENAGATLIQEVAIKMNESAGDGTTTAIILAREMIKAGSLAIAFGAN
Section 3
(157) 157 170 180 190 200 210 220 234
Cpn60alpha1 At2g28000 (157) PVSLKRGIDKTVQGLIEELQKKARPVKGRDDIRAVASISAGNDDLIGSMIADAIDKVGPDGVLSIESSSSFETTVEVE
Cpn60alpha2 At5g18820 (144) AVSVKNGMNKTVKELVRVLQMKSIPVQGKNDIKAVASISAGNDEFVGNLIAETVEKIGPDGVISIESSSTSETSVIVE
Section 4
(235) 235 240 250 260 270 280 290 300 312
Cpn60alpha1 At2g28000 (235) EGMEIDRGYISPQFVTNPEKLLAEFENARVLITDQKITAIKDIIPILEKTTQLRAPLLIIAEDVTGEALATLVVNKLR
Cpn60alpha2 At5g18820 (222) EGMKFDKGYMSPHFITNQEKSTVEFDKAKILVTDQKITSAKELVPLLEKTSQLSVPLLIIAEDISAEVLEILVVNKKQ
Section 5
(313) 313 320 330 340 350 360 370 380 390
Cpn60alpha1 At2g28000 (313) GVLNVVAVKAPGFGERRKAMLQDIAILTGAEYLAMDMSLLVENATIDQLGIARKVTISKDSTTLIADAASKDELQARI
Cpn60alpha2 At5g18820 (300) GLINVAVVKCPGMLDGKKALLQDIALMTGADYLSGDLGMSLMGATSDQLGVSRRVVITANSTTIVADASTKPEIQARI

D

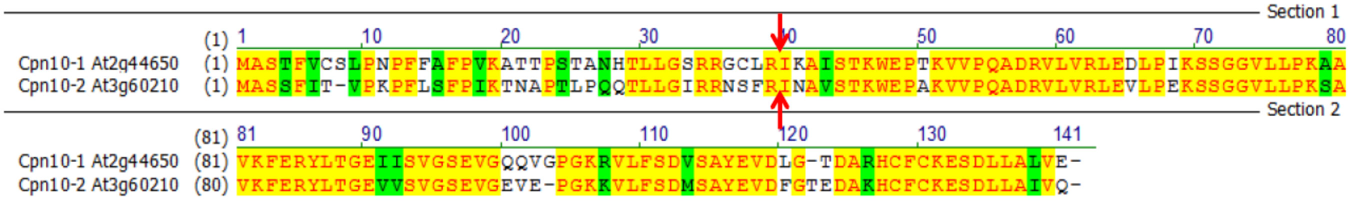
Section 1
(1) 1 10 20 30 40 50 60 70 80
Cpn10-1 At2g44650 (1) MASTFVCSLPNPFFAFPVKATTPSTANHTLLGSRRGCLRIKAISTKWEPTKVVPQADRVLVRLEDLPIKSSGGVLLPKAA
Cpn10-2 At3g60210 (1) MASSFIT-VPKPFLSFPIKTNAPTLPQQTLLGIRRNSFRINAVSTKWEPAKVVPQADRVLVRLEVLPEKSSGGVLLPKSA
Section 2
(81) 81 90 100 110 120 130 141
Cpn10-1 At2g44650 (81) VKFERYLTGEIISVGSEVGQQVGPGKRVLFSDVSAYEVDLG-TDARHCFCKESDLLALVE-
Cpn10-2 At3g60210 (80) VKFERYLTGEVVSVGSEVGEVE-PGKKVLFSDMSAYEVDFGTEDAKHCFCKESDLLAIVQ-

E

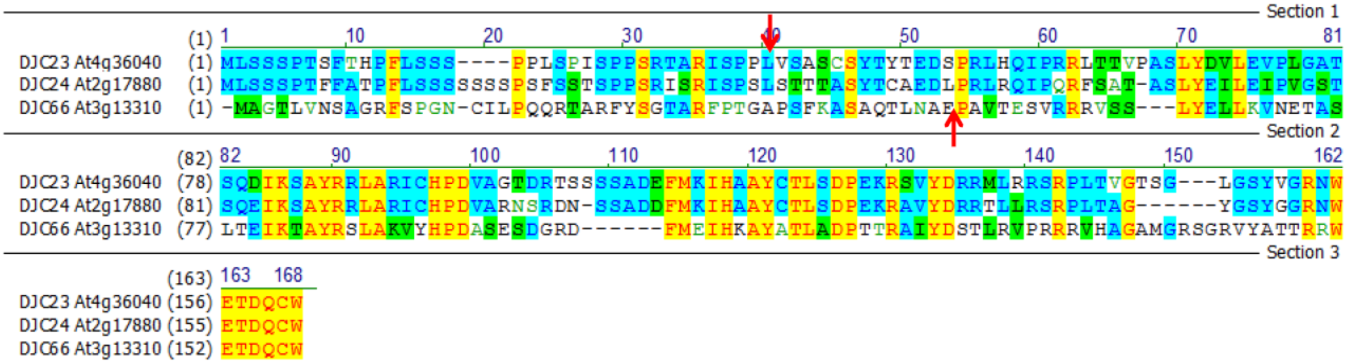
Section 1
(1) 1 10 20 30 40 50 60 70 81
DJC23 At4g36040 (1) MLSSSPTSFTHPFLSSS----PPLSPISPPSRTARISPPLVSASCSYTYTEDSPRLHQIPRRLTTVPASLYDVLEVPLGAT
DJC24 At2g17880 (1) MLSSSPTFFATPFLSSSSSSSPSFSSTSPPSRISRISPSLSTTTASYTCAEDLPRLRQIPQRFSAT-ASLYEILEIPVGST
DJC66 At3g13310 (1) -MAGTLVNSAGRFSPGN-CILPQQRTARFYSGTARFPTGAPSFKASAQTLNAEPAVTESVRRRVSS---LYELLKVNETAS
Section 2
(82) 82 90 100 110 120 130 140 150 162
DJC23 At4g36040 (78) SQDIKSAYRRLARICHPDVAGTDRTSSSSADEFMKIHAAYCTLSDPEKRSVYDRRMLRRSRPLTVGTSG---LGSYVGRNW
DJC24 At2g17880 (81) SQEIKSAYRRLARICHPDVARNSRDN-SSADDFMKIHAAYCTLSDPEKRAVYDRRTLLRSRPLTAG------YGSYGGRNW
DJC66 At3g13310 (77) LTEIKTAYRSLAKVYHPDASESDGRD------FMEIHKAYATLADPTTRAIYDSTLRVPRRRVHAGAMGRSGRVYATTRRW
Section 3
(163) 163 168
DJC23 At4g36040 (156) ETDQCW
DJC24 At2g17880 (155) ETDQCW
DJC66 At3g13310 (152) ETDQCW
