# Supplementary material for: Differential Age-Dependent Import Regulation by Signal Peptides
Source: PLoS Biol. 2012 Oct 30;10(10):e1001416. doi: 10.1371/journal.pbio.1001416 (PMC3484058; doi:10.1371/journal.pbio.1001416)
Supplement: Table S1 — Full names and accession numbers of precursors used in this study. Transit peptide sequences of group III precursors are also included. Two consecutive amino acids with positive charges are underlined. Transit peptide processing site is based on ChloroP prediction or published literature. Two consecutive positive charges are not found within the ChloroP-predicted transit peptide of DJC66 (predicted processing site indicated by an upward arrow), but three consecutive Arg are found eight amino acids downstream. Proteins with accession numbers with “Atxg” are from Arabidopsis. (DOC) [file pbio.1001416.s009.doc]

| **Table S1. Full names and accession numbers of precursors used in this study.** Transit peptide sequences of group III precursors are also included. Two consecutive positive charges are underlined. Transit peptide processing site is based on ChloroP prediction or published literature. Two consecutive positive charges are not found within the ChloroP-predicted transit peptide of DJC66 (predicted processing site indicated by an upward arrow) but three consecutive Arg are found 8 amino acids downstream. Proteins with accession numbers Atxg are from Arabidopsis. | | | |
| --- | --- | --- | --- |
| Group | Name | Full name and other names | Accession number |
| I | RBCS | Small subunit of RuBP carboxylase (a hybrid protein of soybean prRBCS transit peptide and pea prRBCS mature protein [1]) |  |
|  | OE23 | 23-kD protein of the oxygen evolving complex | At1g06680 |
|  | PC | Plastocyanin of pea | X16082 |
|  | DJC22 | Type III J-domain containing protein 22, DnaJ-J8, AtJ8 [2] | At1g80920 |
|  | DJC75 | Type III J-domain containing protein 75, CRRJ [3] (Chlororespiratory reduction J), NdhT [4] (NADH dehydrogenase T), AtJ25 [2] | At4g09350 |
|  | OE33 | 33-kD protein of the oxygen evolving complex | At5g66570 |
|  | Fd-protA | Ferredoxin transit peptide fused to *Staphylococcal* protein A [5] |  |
|  | Cpn60α1 | Chloroplast chaperonin 60α1 subunit | At2g28000 |
|  | Cpn10-1 | Chloroplast chaperonin 10-1 | At2g44650 |
|  | BCCP-2 | Biotin carboxyl carrier protein 2 | At5g15530 |
|  | DJC23 | Type III J-domain containing protein 23, AtJ11 [2] | At4g36040 |
|  | DJC24 | Type III J-domain containing protein 24, AtJ41 [2] | At2g17880 |
|  | Cr-RBCS | Small subunit of RuBP carboxylase of *Chlamydomonas reinhardtii* | AV388873 |
| II | Hsp93 | Heat shock protein 93 kD of pea chloroplasts | L09547 |
|  | PDH | Pyruvate dehydrogenase E1 α subunit | At1g01910 |
|  | Tic20 | Translocon at the inner-envelope-membrane of chloroplasts 20 kD | At1g04940 |
|  | cpHsc70-2 | Chloroplast heat shock protein 70 kD | At5g49910 |
|  | Glu2 | ferredoxin-dependent glutamate synthase 2 | At2g41220 |
|  | Cpn60α2 | Chloroplast chaperonin 60α2 subunit | At5g18820 |
|  | Cpn10-2 | Chloroplast chaperonin 10-2 | At3g60210 |
|  | BCCP-1 | Biotin carboxyl carrier protein 1 | At5g16390 |
| III | Tic40 | Translocon at the inner-envelope-membrane of chloroplasts 40 kD of pea | AY157668 |
|  |  | MENLNLALVS SPKPLLLGHS SSKNVFSG**RK** SFTFGTFRVS ANSSSSHVTR AASKSHQNLK SVQGKVNAHD FA |  |
|  | L11 | L11 subunit of the chloroplast 50S ribosome | At1g32990 |
|  |  | MASSSLSTLC SSTSSSLHPN SKLSHSLSAK LSSKANVSVQ FLG**KK**QSPLL SSTPRFLTVI |  |
|  | cpHsc70-1 | Chloroplast heat shock protein 70 kD | At4g24280 |
|  |  | MASSAAQIHV LGGIGFASSS SS**KR**NLNGKG GTFMPRSAFF GTRTGPFSTP TSAFLRMGTR NGGGASRYAV GPVRVVNEKV VGIDLGTTNS AV |  |
|  | PORA | Protochlorophyllide oxidoreductase A | At5g54190 |
|  |  | MALQAASLVS SAFSV**RK**DGK LNASASSSFK ESSLFGVSLS EQSKADFVSS SLR |  |
|  | PORB | Protochlorophyllide oxidoreductase B | At4g27440 |
|  |  | MALQAASLVS SAFSV**RK**DAK LNASSSSFKD SSLFGASITD QIK |  |
|  | PORC | Protochlorophyllide oxidoreductase C | At1g03630 |
|  |  | MALQAAYSLL PSTISIQKEG KFNASLKETT FTGSSFSNHL RAEKISTLLT IKEQ**RR**QKPR FSTGIRAQTV T |  |
|  | DJC66 | Type III J-domain containing protein 66, AtJ38 [2] | At3g13310 |
|  |  | MAGTLVNSAG RFSPGNCILP QQRTARFYSG TARFPTGAPS FKASAQTLNA↑EPAVTESV**RR R** |  |
|  | Cr-L11 | L11 subunit of the chloroplast 50S ribosome of *Chlamydomonas reinhardtii* | AV390140 |
|  |  | MAAVMPRSGV CLPTRSARLA FAPARVATAN **RK**VTTM |  |

**References**

1. Lubben TH, Keegstra K (1986) Efficient in vitro import of a cytosolic heat shock protein into pea chloroplasts. Proc Natl Acad Sci U S A 83: 5502-5506.

2. Miernyk JA (2001) The J-domain proteins of Arabidopsis thaliana: an unexpectedly large and diverse family of chaperones. Cell Stress Chaperones 6: 209-218.

3. Yamamoto H, Peng L, Fukao Y, Shikanai T (2011) An Src homology 3 domain-like fold protein forms a ferredoxin binding site for the chloroplast NADH dehydrogenase-like complex in Arabidopsis. Plant Cell 23: 1480-1493.

4. Ifuku K, Endo T, Shikanai T, Aro EM (2011) Structure of the chloroplast NADH dehydrogenase-like complex: nomenclature for nuclear-encoded subunits. Plant Cell Physiol 52: 1560-1568.

5. Ma Y, Kouranov A, LaSala SE, Schnell DJ (1996) Two components of the chloroplast protein import apparatus IAP86 and IAP75, interact with the transit sequence during the recognition and translocation of precursor proteins at the outer envelope. J Cell Biol 134: 315-327.
