# Supplementary material for: Differential Age-Dependent Import Regulation by Signal Peptides
Source: PLoS Biol. 2012 Oct 30;10(10):e1001416. doi: 10.1371/journal.pbio.1001416 (PMC3484058; doi:10.1371/journal.pbio.1001416)
Supplement: Table S2 — Primers used for building fusion constructs. The restriction enzyme sites used for cloning are underlined. (DOC) [file pbio.1001416.s010.doc]

**Supplementary Information Table S2. Primers used for building fusion constructs. The restriction** enzyme sites used for cloning are underlined.

| construct | fragment amplified | Primer (5’ -> 3’) |
| --- | --- | --- |
| *RBCStp-mTic40* | prRBCS transit peptide | prSSTP-BamHI-F1(5’-CCCGGATCCATGGCTTCCTCAATGATCTCC)  tpSS-m40-R1(5’-AGAAAATATACTTGCAAAGCATTGTACTCTTCCACC) |
|  | Tic40 mature region | tpSS-m40-F1(5’-GGTGGAAGAGTACAATGCTTTGCAAGTATATTTTCT)  T7 (5’-TAATACGACTCACTATA) |
| *Tic40tp-mRBCS* | prTic40 transit peptide | atTci40TP-XhoI-F1(5’-CCCCTCGAGATGGAGAACCTTACCCTAGTT)  atTci40TP-SphI-R1(5’-CCCGCATGCTAGCAACAGAAGTTGTCTGTT) |
| *RBCStp-GST* | Pea prRBCS transit peptide | PrSS-GST-F (5’-ACC CCT GCA TCA GGG ATA TCC TCT TCA GCT-3’)  PrSS-GST-R (5’-CTT TCC TAT TGG AGG CCA CAC CTG CAT-3’) |
|  | *PsRBCStp-GST* | PrSSTP-GST-F1(5’-GGATCCATGTCCCCTATATCAGGGATATCC)  GST-EcoRI-R1(5’-ACCCGGGAATTCTCAGATCCCACGACC) |
| *Tic40tp-GST* | prTic40 transit peptide | T7 (5’-TAATACGACTCACTATA)  psTic40-SphI-R1(5’-AGAAGAAAGCATGCAAAAATCATGCGCATTCAC) |
| *TIC40p:RBCStp-mTic40* | *TIC40* promoter | P40-KpnI-F1 (5’-CCCGGTACCAGTCTCTGGACATGTAGCATT)  UTR-R1(5’-GATCATTGAGGAAGCCATATCTAATTGTTTCTTTGG) |
|  | *RBCStp-mTic40* | UTR-F1(5’-CCAAAGAAACAATTAGATATGGCTTCCTCAATGATC)  m40-XbaI-R1(5’-CCCTCTAGATCAACCCGTCATTCCTGGGAA) |
| *TIC40p:prTic40* | *TIC40* promoter | P40-KpnI-F1 (5’-CCCGGTACCAGTCTCTGGACATGTAGCATT)  UTR-R2(5’-TAGGGTAAGGTTCTCCATATCTAATTGTTTCTTTGG) |
|  | *prTic40* | UTR-F2(5’-CCAAAGAAACAATTAGATATGGAGAACCTTACCCTA)  m40-XbaI-R1(5’-CCCTCTAGATCAACCCGTCATTCCTGGGAA) |
| prTic40-A(2-9) | Ala substitutions from residue 2 to 9 of prTic40 transit peptide | Tic40A(2-9)F(5’-GGTATCGATAAGCTTGATATGGCGGCTGCTGCCGCAGCCGCTGCTTCTTCCCCTAAACCCCTG)  Tic40A(2-9)R(5’-  CAGGGGTTTAGGGGAAGAAGCAGCGGCTGCGGCAGCAGCCGCCATATCAAGCTTATCGATACC) |
| prTic40-A(10-18) | Ala substitutions from residue 10 to 18 of prTic40 transit peptide | Tic40A(10-18)F(5’-  CTTAACTTAGCCCTTGTTGCTGCCGCTGCAGCCGCGGCTGCAGCACATTCCTCCTCAAAAAAC)  Tic40A(10-18)R(5’-  GTTTTTTGAGGAGGAATGTGCTGCAGCCGCGGCTGCAGCGGCAGCAACAAGGGCTAAGTTAAG) |
| prTic40-A(19-27) | Ala substitutions from residue 19 to 27 of prTic40 transit peptide | A(19-27)F(5’-AAACCCCTGCTTTTAGGAGCTGCCGCCGCAGCAGCCGCTGCCGCAGGAAGGAAGTCTTTCACT)  A(19-27)R(5’-  AGTGAAAGACTTCCTTCCTGCGGCAGCGGCTGCTGCGGCGGCAGCTCCTAAAAGCAGGGGTTT) |
| prTic40-A(28-36) | Ala substitutions from residue 28 to 36 of prTic40 transit peptide | Tic40A(28-36)F(5’-  TCAAAAAACGTTTTCTCAGCAGCGGCGGCTGCCGCTGCTGCGGCGTTTCGCGTTTCTGCTAAC)  Tic40A(28-36)R(5’-  GTTAGCAGAAACGCGAAACGCCGCAGCAGCGGCAGCCGCCGCTGCTGAGAAAACGTTTTTTGA) |
| prTic40-A(37-45) | Ala substitutions from residue 37 to 45 of prTic40 transit peptide | Tic40A(37-45)F(5’-  TCTTTCACTTTTGGGACGGCTGCCGCTGCTGCTGCCGCTGCAGCCTCTCATGTCACCAGGGCT)  Tic40A(37-45)R(5’-  AGCCCTGGTGACATGAGAGGCTGCAGCGGCAGCAGCAGCGGCAGCCGTCCCAAAAGTGAAAGA) |
| prTic40-A(46-54) | Ala substitutions from residue 46 to 54 of prTic40 transit peptide | Tic40A(46-54)F(5’-  TCTGCTAACTCTTCATCCGCTGCTGCCGCCGCGGCTGCTGCTGCATCTCACCAAAATCTAAAA)  Tic40A(46-54)R(5’-  TTTTAGATTTTGGTGAGATGCAGCAGCAGCCGCGGCGGCAGCAGCGGATGAAGAGTTAGCAGA) |
| prTic40-A(55-63) | Ala substitutions from residue 55 to 63 of prTic40 transit peptide | Tic40A(55-63)F(5’-  ACCAGGGCTGCTTCTAAAGCTGCCGCAGCTGCAGCAGCTGCGGCGGGGAAGGTGAATGCGCAT)  Tic40A(55-63)R(5’-  ATGCGCATTCACCTTCCCCGCCGCAGCTGCTGCAGCTGCGGCAGCTTTAGAAGCAGCCCTGGT) |
| prTic40-A(64-72) | Ala substitutions from residue 64 to 72 of prTic40 transit peptide | Tic40A(64-72)F(5’-  AATCTAAAATCTGTGCAGGCGGCGGCGGCTGCGGCTGCTGCTGCTAGCATTTCTTCTTCAAAT)  Tic40A(64-72)R(5’-  ATTTGAAGAAGAAATGCTAGCAGCAGCAGCCGCAGCCGCCGCCGCCTGCACAGATTTTAGATT) |
| prTic40-A(28-31) | Ala substitutions from residue 28 to 31 of prTic40 transit peptide | Tic40A(28-31)F(5’-  TCAAAAAACGTTTTCTCAGCAGCGGCGGCTTTCACTTTTGGGACG TTT)  Tic40A(28-31)R(5’-  AAACGTCCCAAAAGTGAAAGCCGCCGCTGCTGAGAAAACGTTTTT TGA) |
| prTic40-A(32-36) | Ala substitutions from residue 32 to 36 of prTic40 transit peptide | Tic40A(32-36)F(5’-  TTCTCAGGAAGGAAGTCTGCCGCTGCTGCGGCGTTTCGCGTTTCT GCTAAC)  Tic40A(32-36)R(5’-  GTTAGCAGAAACGCGAAACGCCGCAGCAGCGGCAGACTTCCTTCCTGAGAA) |
| prTic40(G28P) |  | Tic40(G28P)F(5’-  CTCAAAAAACGTTTTCTCACCAAGGAAGTCTTTCACTTTTGG)  Tic40(G28P)F(5’-  CCAAAAGTGAAAGACTTCCTTGGTGAGAAAACGTTTTTTGAG) |
| prTic40(S31A) |  | Tic40(S31A)F(5’-  CGTTTTCTCAGGAAGGAAGGCTTTCACTTTTGGGACGTTTCGC)  Tic40(S31A)F(5’-  GCGAAACGTCCCAAAAGTGAAAGCCTTCCTTCCTGAGAAAACG) |
| prTic40(R29E) |  | Tic40(R29E)F(5’-  CAAAAAACGTTTTCTCAGGAGAGAAGTCTTTCACTTTTGGGACG)  Tic40(R29E)R(5’-  CGTCCCAAAAGTGAAAGACTTCTCTCCTGAGAAAACGTTTTTTG) |
| prTic40(K30E) |  | Tic40(K30E)F(5’-  CAAAAAACGTTTTCTCAGGAAGGGAGTCTTTCACTTTTGGGACG)  Tic40(K30E)R(5’-  CGTCCCAAAAGTGAAAGACTCCCTTCCTGAGAAAACGTTTTTTG) |
| prTic40(RK2930EE) |  | Tic40(RK2930EE)F(5’-  CAAAAAACGTTTTCTCAGGAGAGGAGTCTTTCACTTTTGGGACG)  Tic40(RK2930EE)R(5’-  CGTCCCAAAAGTGAAAGACTCCTCTCCTGAGAAAACGTTTTTTG) |
| prTic40(RK2930AA) |  | Tic40(RK2930AA)F(5’-  CAAAAAACGTTTTCTCAGGAGCGGCGTCTTTCACTTTTGGGACG)  Tic40(RK2930AA)R(5’-  CGTCCCAAAAGTGAAAGACGCCGCTCCTGAGAAAACGTTTTTTG) |
| prTic40-A(28-31)+RK |  | Tic40-[A(28-31)+RK]F(5’-  CCTCAAAAAACGTTTTCTCAGCACGGAAGGCTTTCACTTTTGGG ACGTTTCG)  Tic40-[A(28-31)+RK]R(5’-  CGAAACGTCCCAAAAGTGAAAGCCTTCCGTGCTGAGAAAACGTT TTTTGAGG) |
| prTic40-A(28-36)+RK |  | Tic40-[A(28-36)+RK]F(5’-  CCTCAAAAAACGTTTTCTCAGCACGGAAGGCTGCCGCTGCTGCG GCGTTTCG)  Tic40-[A(28-36)+RK]F(5’-  CGAAACGCCGCAGCAGCGGCAGCCTTCCGTGCTGAGAAAACGTT TTTTGAGG) |
| prL11(KK4445EE) |  | L11(KK4445EE)F(5’- CCGTCCAATTTCTGGGAGAGGAACAGTCTCCGCTTCTCTCCTC)  L11(KK4445EE)R(5’-  GAGGAGAGAAGCGGAGACTGTTCCTCTCCCAGAAATTGGACGG) |
